# NarJ subfamily system specific chaperone diversity and evolution is directed by respiratory enzyme associations

**DOI:** 10.1186/s12862-015-0412-3

**Published:** 2015-06-12

**Authors:** Denice C. Bay, Catherine S. Chan, Raymond J. Turner

**Affiliations:** Department of Biological Sciences, University of Calgary, Rm 156 Biological Science Bldg., 2500 University Dr. NW, Calgary, T2N 1 N4 AB Canada

**Keywords:** Redox Enzyme Maturation Protein (REMP), System specific chaperones, NarJ, NarW, DmsD, TorD, YcdY, Complex iron-sulfur molybdoenzymes, Anaerobic respiration, Chaperone, Twin Arginine Translocase (Tat)

## Abstract

**Background:**

Redox enzyme maturation proteins (REMPs) describe a diverse family of prokaryotic chaperones involved in the biogenesis of anaerobic complex iron sulfur molybdoenzyme (CISM) respiratory systems. Many REMP family studies have focused on NarJ subfamily members from *Escherichia coli*: NarJ, NarW, DmsD, TorD and YcdY. The aim of this bioinformatics study was to expand upon the evolution, distribution and genetic association of these 5 REMP members within 130 genome sequenced taxonomically diverse species representing 324 Prokaryotic sequences. NarJ subfamily member diversity was examined at the phylum-species level and at the amino acid/nucleotide level to determine how close their genetic associations were between their respective CISM systems within phyla.

**Results:**

This study revealed that NarJ members possessed unique motifs that distinguished Gram-negative from Gram-positive/Archaeal species and identified a strict genetic association with its nitrate reductase complex (*narGHI*) operon compared to all other members. NarW appears to be found specifically in Gammaproteobacteria. DmsD also showed close associations with the dimethylsulfoxide reductase (*dmsABC*) operon compared to TorD. Phylogenetic analysis revealed that YcdY has recently evolved from DmsD and that YcdY has likely diverged into 2 subfamilies linked to Zn- dependent alkaline phosphatase (*ycdX*) operons and a newly identified operon containing part of Zn-metallopeptidase FtsH complex component (*hflC*) and NADH-quinone dehydrogenase (*mdaB*). TorD demonstrated the greatest diversity in operon association. TorD was identifed within operons from either trimethylamine-N-oxide reductase (*torAC*) or formate dehydrogenase (*fdhGHI*), where each type of TorD had a unique motif. Additionally a subgroup of *dmsD* and *torD* members were also linked to operons with biotin sulfoxide (*bisC*) and polysulfide reductase (*nrfD*) indicating a potential role in the maturation of diverse CISM.

**Conclusion:**

Examination of diverse prokaryotic NarJ subfamily members demonstrates that the evolution and genetic association of each member is uniquely biased by its CISM operon association.

**Electronic supplementary material:**

The online version of this article (doi:10.1186/s12862-015-0412-3) contains supplementary material, which is available to authorized users.

## Background

Anaerobic respiratory enzymes found in a variety of facultative and anaerobic Bacteria and Archaea offer an organism the flexibility to survive in a wide variety of anoxic environments. For example, the facultative Gammaproteobacterial anaerobe *Escherichia coli* has a total of 23 respiratory enzymes ranging from reductases, hydrogenases, dehydrogenases, and oxidases conferring respiratory flexibility and diversity under anaerobic growth conditions (reviewed by [1]). The majority of anaerobic respiratory systems have similar modular cofactor-based protein architecture (as reviewed by [2]). Despite their architectural similarities these systems demonstrate incredible versatility in substrate utilization, reflecting their essential role in energy generation during the first billion years of life [3–5]. Characterized members are generally classified into families and superfamilies according to their substrate(s) and/or co-factor requirements [2]. One of these members is the complex iron-sulfur molybdoenzyme (CISM) superfamily of oxidoreductases, which are typified by the presence of an iron-sulfur cluster and an additional molybdenum-*bis* pyranopterin guanine dinucleotide) (Mo*bis*PGD) catalytic cofactor in its active site [6]. Well-characterized examples from this superfamily include enzyme subfamilies such as dimethylsulfoxide (DMSO) reductase, trimethylamine N-oxide (TMAO) reductase, formate dehydrogenase (Fdh), and nitrate reductase (Nar).

Due to their inherent complexity and cofactor requirements, the biogenesis of any CISM requires the aid of a specialized chaperone known as a redox enzyme maturation protein (REMP). In general, a specific REMP is involved in the maturation of a particular molybdoenzyme(s) [7–9]. Key functions of REMPs include CISM folding, cofactor insertion, membrane/translocase targeting, subunit assembly, proofreading, and proteolytic protection of its respective enzyme during its maturation. The majority of studies on REMPs focus on members identified from the model organism *E. coli*, however, REMPs from other Bacterial and Archaeal species have been functionally and structurally examined (as reviewed by [9, 10]). A distinct subgroup within REMP family members demonstrated high sequence homology and its members include: the Nar delta subunit of the cytoplasmic nitrate reductase A (NarJ), and its closely related homologue (NarW) involved in nitrate reductase Z assembly, DMSO reductase maturation protein D (DmsD), the chaperone protein for trimethylamine-N-oxide oxidoreductase 1 (TorD), and the chaperone protein involved in maturation of YcdX (YcdY) [7, 9, 11]. Other distantly related REMP family members that are not considered part of this subgroup include, FdhD and FdhE affecting formate dehydrogenase-N complex activity [12, 13], HyaE for hydrogenase-1 maturation [14], chaperone for the periplasmic nitrate reductase (NapD) [15], and a hydrogenase-2-specific chaperone (HybE) [14, 16]. This subfamily is known by a variety of names all based on their homology to characterized members such as the DmsD/TorD and/or TorD subfamily [11, 17] and to NarJ [9]. Since this study will specifically focus on characterized members NarJ/NarW, DmsD, TorD and YcdY, the subfamily will be referred to as the NarJ subfamily based on most recent bioinformatics analyses [9] and findings presented in this study.

Every REMP member is responsible for the maturation of a specific enzyme complex by coordinating co-factor insertion, folding, and targeting the holoenzyme to the twin-arginine translocase (TAT) for secretion across the cytoplasmic membrane and attachment to its membrane anchor subunit (as reviewed by [10, 18]). NarJ is the REMP for the cytoplasmic nitrate reductase (NarGHI) and binds the α-subunit NarG [19–21]. NarW is the purported REMP for a second cytoplasmic nitrate reductase complex (NarVYZ) present in some species and *in vitro* studies suggest that it can interact with both NarG and NarZ [22]. Both *narJ* and *narW* are located within the operons of their respective respiratory enzyme substrates (*narGHJI* and *narZYWV*) in *E. coli* [22]. NarJ is also known to interact with Mo*bis*PGD biosynthesis proteins (MogA, MoeA, MobAB) in addition to NarG [21].

DmsD, also known as YnfI, binds the α-subunit (DmsA) of the DMSO reductase (DmsABC) [23–25] and binds to the α-subunits, YnfE and YnfF, of the selenate reductase complex (YnfEFGH) [16, 26] during their maturation. DmsD has demonstrated binding to the preprotein form of TorA and its signal peptide [16, 24]. In *E. coli* the *dmsD/ynfI* locus is part of the *ynfEFGHI* operon and the *dmsABC* operon is located in *trans* from the *ynfEFGHI* operons [27]. DmsD appears to recognize and interact with a variety signal/ leader peptides, general chaperones (GroEL, DnaK, DnaJ, GrpE, Tig and EF-TU), and TAT components [28] suggesting that DmsD acts as a chaperone node in many CISM maturation pathways.

TorD is known to assist in the folding and recognition of the signal peptide of the periplasmic catalytic subunit (TorA) of the TMAO reductase complex 1 (TorAC) [16, 29, 30] and its gene is located within the *torAC* operon in *E. coli* [31]. Unlike DmsD, TorD interacts exclusively with the signal/ leader peptide of the TorA [30, 32]. TorD also possesses a TAT-signal proofreading ability [30, 32, 33] and it was demonstrated to possess GTPase activity [34].

Relatively little is known about YcdY, and it is considered to be an atypical member of the NarJ subfamily due to its involvement in the maturation of a Zn^2+^ cofactor requiring phosphatase (*ycdX*) that participates in swarming motility [35]. In *E. coli, ycdY* mutants result in swarming motility defects and it is not associated with any known CISM systems [35, 36].

The purpose of this bioinformatic study was to examine the sequence distribution, diversity and evolution of all 5 characterized NarJ subfamily REMP members, NarJ, NarW, DmsD, TorD and YcdY within Bacterial and Archaeal kingdoms. The goal was to identify sequence similarities and differences among NarJ subfamily members homologues from a broad taxonomic sampling of 130 diverse Bacterial and Archaeal species (a total of 324 sequences) to gain additional insights into the role(s) that each member of this REMP subgroup may contribute to the maturation of their specific CISM. A combination of bioinformatics approaches were used to examine and compare the distribution, sequence structure and location of NarJ subfamily members and their specific CISM enzymes. These approaches involved operon mapping, phylogenetic analysis of protein sequences, non-synonymous to synonymous nucleotide substitution assessments, and multiple sequence alignments for motif analyses. The main finding revealed that NarJ subfamily evolution was tightly linked to its CISM operon association. A second major finding from this identified the presence of unique subfamily member specific motifs in each chaperone that were linked to different CISM operons. A final novel finding from this study indicates that YcdY recently evolved from DmsD and has diverged into 2 subgroups, one associated with Zn- dependent *ycdX* and a second newly identified subgroup linked to operons containing *hflC* (a ATP dependent Zn-metallopeptidase) and to NADH dehydrogenase (quinone) homologue *mdaB*.

## Methods

### NarJ subfamily nucleotide and protein sequence dataset collection

NarJ subfamily protein and nucleotide sequences analyzed in this study were collected from the completed Archaeal and Bacterial genome sequences available from GenBank on the National Center of Biotechnology Information (NCBI) website (http://www.ncbi.nlm.nih.gov/). NarJ subfamily protein sequences present in microbial genomes were identified using tBLASTn [37] searches, with one of the following *Escherichia coli* NarJ subfamily protein query sequences: NarJ (BAL38294), NarW (AAC74548), TorD (AAC74083), DmsD (AAC74663), and YcdY (AAC74119). A total of 1415 identified NarJ subfamily proteins were detected in 918 Bacterial (782) and Archaeal (136) species using tBLASTn genome searches that were restricted to an e-value cutoff of ≤ 1 × 10^−4^. All 1415 NarJ subfamily protein sequences from tBLASTn searches were reduced to a final total of 324 for examination in this study, representing 130 Bacterial and Archaeal species, using the web-based BLASTClust program (http://toolkit.tuebingen.mpg.de/blastclust). This reduction focused on maintaining the most species diversity within all phyla, while keeping sequence totals at a manageable amount for further bioinformatics analyses. This selection included species with greatest number of NarJ subfamily members. Corresponding nucleotide sequences for each of the 324 protein sequences were obtained and locus tag numbers were used to collect nearby open reading frame (ORF) annotation information from all genes present within a −/+ 10 ORF radius relative to the chaperone locus. A list of all 324 NarJ subfamily sequences examined in this study, their corresponding protein accession numbers, and locus tags are provided in Additional file 1 (Table S1).

### NarJ subfamily protein and nucleotide multiple sequence alignments

Multiple sequence alignments (MSA) of the 324 NarJ subfamily protein sequence dataset were performed prior to bioinformatics analysis. MSA were generated using two programs, the constraint-based online Cobalt software [38] and PRALINE software [39] (http://www.ibi.vu.nl/programs/pralinewww). Both MSA were merged to construct a final NarJ subfamily consensus MSA. The advantage of combining MSA from both methods permitted hidden Markov matrix analysis and neural network predictions to be performed with conserved domain and secondary structure estimations which aided the alignment of many NarJ subfamily sequences with low sequence identity (<10 %). Poorly aligned residues within amino (N)- and carboxyl (C)- termini and within predicted loop/turn regions were trimmed from MSA to improve downstream bioinformatics analyses using the multiple alignment editing program Jalview [40]. Jalview was used to calculate amino acid percentage identities (% identity) and to provide an overall residue consensus at each alignable position within protein and codon nucleotide MSA (Additional file 2: Figure S1). This main 324 protein MSA was subdivided into 5 sub-alignments, generating NarJ, NarW, DmsD, TorD and YcdY protein alignment datasets.

### Phylogenetic analysis of NarJ subfamily proteins

Phylogenetic analysis of the 324 NarJ subfamily sequence dataset was performed using Bayesian inference (BI) with MrBayes version 3.2.1 software [41]. Additional phylogenetic analyses were performed using the neighbour joining (NJ) and maximum likelihood (ML) methods available from the PHYLIP software version 3.695 package [42]. Different methods were used to confidently estimate the evolutionary origins of each subfamily member and validate the relationships of putatively annotated and experimentally characterized members. Both NJ and ML dendrograms showed similar cladistic and branching associations among all the NarJ subfamily sequences as observed for BI and were used to validate the cladistic outcomes for BI. BI was selected to generate all dendrograms used for this study since it provided the highest posterior probability (PP) confidence values at all major nodes as compared to the bootstrapped values performed by NJ and ML [43]. Phylogenetic analyses were performed using a variety of Crenarchaeal NarJ subfamily homologues of NarJ, TorD and DmsD as outgroups. Multiple Crenarchaeal NarJ subfamily sequences were used as outgroups (either *Vulcanisaeta distributa* NarJ, *Thermoproteus tenax* TorD or *Vulcanisaeta distributa* DmsD) for all 3 phylogenetic methods and produced dendrograms with similar cladistic outcomes. Only the Crenarchaeal *V. distributa* NarJ sequence produced dendrograms with the lowest branch polytomy and this sequence was selected for this study. The use of other Archaeal REMP family maturases/ chaperones as outgroups, such as the Fdh formation protein D/E (FdhE and/or FdhD), the signal peptide-binding chaperone for periplasmic nitrate reductase protein A (NapD) and the chaperone for the β subunit of hydrogenase isoenzyme protein E (HyaE), all resulted in dendrograms with unresolvable and extremely polychotomous branching and were not considered further. In an attempt to reduce polytomy occurrence, phylogenetic analyses were performed using Crenarchaeal NarJ, DmsD or TorD sequences as outgroups. Comparison between phylograms, where one of the 3 Crenarchaeal NarJ subfamily members served as an outgroup, determined that the Crenarchaeal NarJ outgroup produced dendrograms with the greatest confidence values (BI was superior) at major nodes and dendrograms with the least polytomy (Additional file 3: Figure S3). As a result, the BI dendrogram using the Crenarchaeal NarJ outgroup was selected for final analysis in this study (Fig. 3).

The BI dendrogram of the 324 NarJ subfamily sequence dataset required a total of 10 million generations using 8 chains at standard burn-in rate where 25 % of the samples were discarded and the tree space was sampled every 1000 generations. Model jumping between fixed-rate amino acid models was used to determine the most suitable substitution model. Mixed model BI analysis favoured either the Jones Taylor Thornton (JTT) [44] or the Blocks of substitution matrix 62 (Blosum62) substitution model [45]. BI convergence was assessed by monitoring the average standard deviation of the split frequencies which was 0.023 at the end of the analysis. BI PP values were calculated using Markov chain Monte Carlo sampling (MCMC) to determine the confidence at each branch nodes [41]. BI dendrogram of the final 324 NarJ subfamily protein sequence dataset is provided in Fig. 3 as an unrooted tree and the rooted NJ dendrogram in Additional file 4: Figure S4.

### NarJ subfamily and CISM respiratory enzyme operon analysis

Operon analysis was performed for each of the 324 NarJ subfamily sequences by examining the first 10 annotated ORFs (upstream and downstream) from each member’s locus tag. This analysis was combined with NarJ subfamily member distributions and CISM operon surveys to determine if each member was located *cis* (within the CISM operon) or *trans* (not included in the CISM operon) to their respective CISM loci. The frequency of occurrence for a particular gene at all NarJ subfamily member loci and other ORF/genes that were identified at *narJ*, *torD*, *dmsD*, and *ycdY* loci were calculated by the number of times its gene name, domain tag, and/ or by its enzyme name was identified from gene annotations. The frequency of gene occurrence (F^G^), was determined by the equation [F^G^ = (n^gene^/ n^locus^) / O^NarJ^] where n^gene^ is the number of times a gene is identified within a ten gene radius from the specific subfamily member’s locus, n^locus^ is the number of total NarJ subfamily loci examined, and O^NarJ^ is total number of NarJ subfamily member operons examined. The reading frames of all genes were determined for each operon and their proximity to the NarJ subfamily locus as summarized in Fig. 2. The outcome of this analysis was used as a guide to subgroup regions within the NarJ subfamily MSA (Additional file 2: Figure S1).

The presence and number of CISM respiratory genes present in each of the 130 species selected for this survey was determined using the NCBI Gene online database (http://www.ncbi.nlm.nih.gov/gene/). Nar, Dms, Tor and Ycd CISM genes were identified using the search terms ‘nitrate reductase’, ‘dimethylsulfoxide/ DMSO reductase’, ‘trimethylamine N-oxide (TMAO) reductase’, ‘polymerase/ histidinol alkaline phosphatase’, ‘ycdX’. Species lacking any of these 4 reductases and YcdX phosphatase were confirmed using tBLASTn genome searches with *E.coli* CISM protein sequences of the missing reductase/ phosphatase with e-value cutoffs of ≤ 1 ×10^−4^. To determine the presence of additional CISM oxidoreductases/ dehydrogenases NCBI gene searches were performed for all 130 species using the search terms ‘molybdopterin’ and one of the following terms ‘reductase’, ‘oxidoreductase’ or ‘dehydrogenase’. Locus tags for all CISM genes were collected and statistically assessed using the ‘R’ statistics software package (http://www.r-project.org/) heatmap and clustering *heatmap.2* function.

In some cases, tBLASTn searches for CISM genes were performed to confirm the presence or absence of CISM genes/operons within the 130 selected species. This search was performed specifically for species lacking annotated CISM genes, like *torAC*. In these cases, tBLASTn searches were conducted using *E. coli* TorA b0997 and TorC b0996 as seed sequences. Species lacking *torAC* were only confirmed when tBLASTn cutoffs were well above acceptable e-value cutoff values ≥ 1.0 × 10^−2^.

### Non-synonymous to synonymous nucleotide substitution analyses of NarJ subfamily sequences

Non-synonymous to synonymous nucleotide substitution analyses of NarJ subfamily sequences were performed with synonymous non-synonymous analysis program (SNAP) package [46] (http://hcv.lanl.gov/content/sequence/SNAP/perlsnap.html). Prior to this analysis, a codon alignment of all 324 NarJ subfamily nucleotide sequences was generated from the corresponding protein MSA (Additional file 2: Figure S1) using the program Mesquite version 2.75 [47] (http://mesquiteproject.org). SNAP analysis of all 324 NarJ subfamily codon aligned sequences provided total synonymous (Sd) and non-synonymous (Nd) codon substitution values which were used to determine substitution rates at each codon position across the entire sequence. The number of observed synonymous nucleotide substitutions (Sd) at each aligned codon position for each member of the NarJ subfamily is provided in Additional file 5: Figure S5. Sd and Nd values were used to calculate the proportion of synonymous (pS) and non-synonymous (pN) substitutions used to determine the non-synonymous to synonymous nucleotide substitution rate (dN/dS) for the entire protein [48]. All dN/dS values were calculated from all pairwise sequence comparisons of codon aligned NarJ subfamily members from the same subgroup (ie. *narJ* to *narJ*). In some cases, pairwise dN/dS values could not be determined due to pS values exceeding the Jukes-Cantor cutoff of 0.75 [48]. dN/dS values for determined each of the 5 NarJ subfamily members were used to calculate percentiles used to generate plots provided in Fig. 4. In some cases, NarJ subfamily member sequences exceeded the Jukes-Cantor distance correction used for these analyses [48] preventing their dN/dS value calculations within certain phyla.

## Results

### NarJ subfamily members and their respective CISM distributions vary within Bacterial and Archaeal phyla

**Fig. 1:**
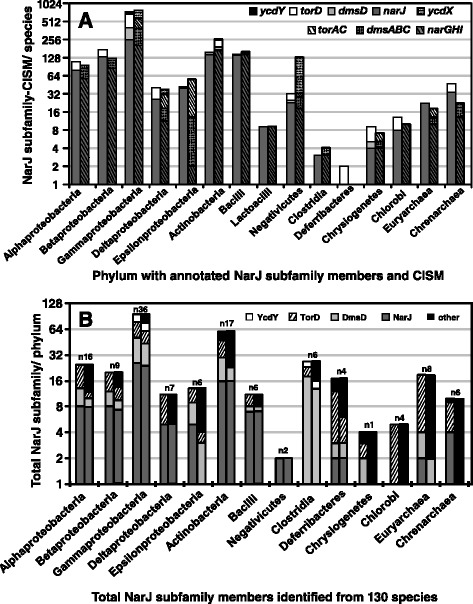
A summary of total annotated and verified NarJ subfamily member-CISM distributions within Prokaryotic species examined in this study. In panel A the left-hand bars indicate all Bacterial and Archaeal species according to phylum with one or more annotated NarJ subfamily members prior to analysis in this study. The right-hand bars in panel **a** indicate all Bacterial and Archaeal species (according to phylum) that possess one or more annotated respiratory enzyme operons prior to performing any analysis. In panel **b**, the left- hand bars represents the total number of annotated subfamily members per phylum and the right-hand bars represents the total number of annotated members per phylum that associated with their respective CISM operon. In both panels the y-axis scale of total NarJ subfamily member values is shown as a log_2_ value. The x-axis in both panels lists all phylum (and in the case of Firmicutes the classes, Bacilli, Lactobacilli, and Clostridia) with identifiable NarJ subfamily homologues

Initial surveys examining the association of NarJ subfamily member to the presence of its respective CISM operon based on genome annotation determined that each subfamily member was ≥ 1 CISM in the genomes of representative phyla (Fig. 1a). This ratio indicated that most NarJ subfamily members, in particular *dmsD* and *ycdY*, were either underrepresented or possibly misannotated in annotated genomes for most species. Annotated *narJ*-*narGHI* demonstrated the most consistent identification ratios within almost all surveyed phyla. Annotated NarW were only identified in 4 Gammaproteobacterial species indicating that these subfamily members have a highly specific distribution. Higher ratios of *torD* to *torAC* operons were detected within Gram-negatives specifically suggesting that many *torD* sequences may be misannotated and appear to lack *torAC* (Fig. 1a).

**Fig. 2:**
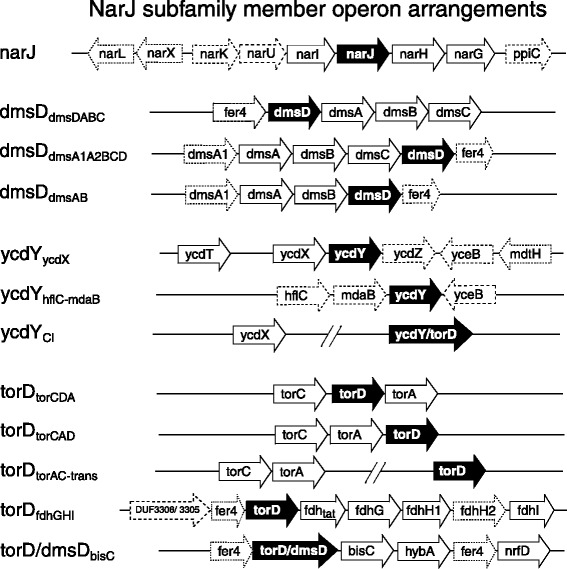
A summary of NarJ subfamily member operon associations and genetic arrangements. A diagrammatic summary of genes frequently co-occurring within a 10 ORF radius of NarJ subfamily member (*narJ*, *dmsD*, *ycdY* and *torD*) loci and operons are shown as labelled arrows. All identified ORFs/genes (labelled according to three letter gene abbreviations) are provided in their order of occurrence from each indicated locus (left-hand side). Solid lined unfilled arrows indicate ORF present at ≥80 % co-frequency occurrence and dashed lined unfilled arrows indicate ORF at ≤ 60 % co-frequency of occurrence to NarJ subfamily member loci (black filled arrows). ORF abbreviations listed in each arrow are as follows: biotin sulfoxide reductase molybdoenzyme family (*bisC*), DMSO reductase alpha molybdoenzyme containing subunit (*dmsA/ dmsA1*), DMSO reductase beta iron-sulfur subunit (*dmsB*), DMSO reductase membrane anchor subunit (*dmsC*), 200 amino acid protein of unknown function (DUF3306), 170–190 amino acid protein of unknown function (DUF3305), TAT pathway signal sequence domain-containing protein (*fdh*_*ta*t_), Fdh-N alpha molybdoenzyme subunit G (*fdhG*), fdh-N beta iron-sulfur subunits H1/H2 (*fdhH1/fdhH2*), Fdh-N gamma membrane anchor subunit I (*fdhI*), 4Fe-4S binding domain containing protein (*fer4*), FtsH protease membrane subunit (*hflC*), 4Fe-4S-cluster-containing hydrogenase component/ 4Fe-4S ferredoxin (*hybA*), NADPH-quinone reductase/ dehydrogenase (*mdaB*), nitrate reductase alpha molybdoenzyme subunit (*narG*), nitrate reductase beta iron-sulfur subunit (*narH*), nitrate reductase gamma membrane anchor subunit (*narI*), nitrate/ nitrite major facilitator transporter (*narK*), nitrate/ nitrite major facilliator transporter (*narU*), polysulfide reductase/ formate-dependent nitrite reductase membrane component (*nrfD*), major facilitator multidrug resistance transporter (*mdtH*), parvulin-like peptidyl-prolyl isomerase (*ppiC*), TMAO reductase I catalytic subunit (*torA*), TMAO reductase c-type cytochrome (*torC*), two-component regulatory system sensory histidine kinase (*narX*), response regulator of two-component regulatory system (*narL*), membrane-anchored diguanylate cyclase (*ycdT*), Zinc- binding alkaline phosphatase/ hydrolase (*ycdX*), 5 transmembrane inner membrane protein of unknown function DUF1097 (*ycdZ*), unknown lipoprotein DUF1439/ PRK10598 (*yceB*)

**Fig. 3:**
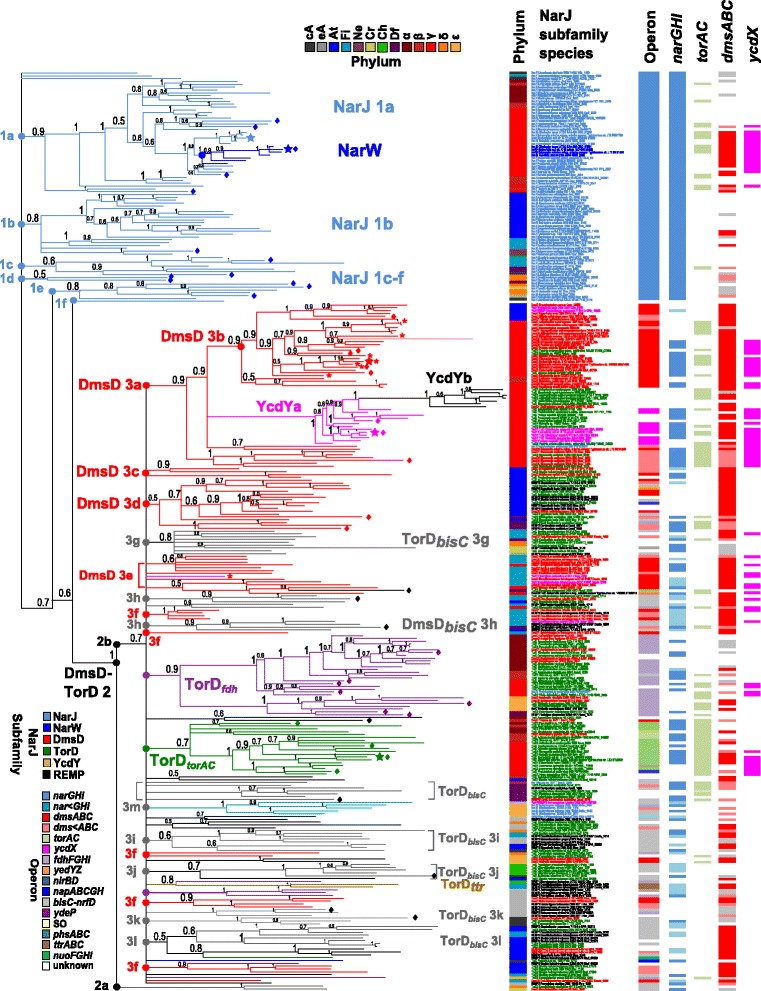
Phylogenetic analysis of Bacterial and Archaeal NarJ, NarW, DmsD, TorD and YcdY sequences. A rooted BI phylogram of 324 protein sequences from 130 different Bacterial and Archaeal species was performed using the Crenarchaeal *Vulcanisaeta distributa* NarJ sequence as an outgroup. A total of 10 million generations were performed and posterior probability values (0–1) at each node of the dendrogram are shown to provide branch confidence. Species (including Genbank locus tag) are listed on the right of each branch and coloured according to its NarJ subfamily member annotation (NarJ blue; DmsD red; TorD green; YcdY pink). Clades (1–3) and subclades (lettered) of significance that are discussed in the results section are highlighted by coloured circles; NarJ (1), DmsD (2) and TorD (3). The coloured heatmap to the right of species labels indicates the operon association of the NarJ subfamily member (operon). Starred branches indicate NarJ subfamily sequences from *E.coli*. Branches with asterisks identify DmsD associated with an *ynfEFGH* operon and branches with diamonds indicate NarJ subfamily sequences with transposon/ integrase genes identified within the +/− 10 ORF region of the operon

To validate associations between each NarJ subfamily member and its specific CISM, the genetic arrangement of each NarJ subfamily member with respect to its operon and a survey of their abundance and distribution within 130 taxonomically diverse species representing each phyla know to possess subfamily members were performed (Fig. 1b). Based on this analysis, *narJ* –*narGHI* operon associations showed the least difference in phylum distribution when compared to their sequence annotations (Fig. 1a and b) and *narJ* demonstrated the highest *cis* conservation with the *narGHI* operon in almost all surveyed phyla (Figs. 1b and 2). In contrast to annotation surveys, *torD-torAC* was identified primarily in Gram-negative proteobacteria and *torD-torAC* was absent in almost all Gram-positives (except Negativicutes) and all Archaeal phyla (Fig. 1a and b). Only 1/3 of all annotated *torD* sequences had any association (both *cis* and *trans*) with its respective *torAC* operon (Fig. 1b and Additional file 6: Figure S2). The remaining 2/3, annotated as ‘TorD’ and/or “nitrate reductase delta”, lacked a detectable *torAC* operon based on tBLASTn searches. Verified *ycdY* –*ycdX* sequence distribution was restricted primarily to Gammaproteobacteria and to a lesser extent in 3 of 6 species Clostridial and 3 Actinobacterial species (*Amycolatopsis orientalis* HCCB10007, *Olsenella uli* DSM 7084 and *Coriobacterium glomerans* PW2) (Fig. 1b). Closer inspection of DmsD indicated that one or more copies of *dmsD*/*ynfI* were located in a *dmsABC/ ynfEFGH* operon confirming that *dmsD* was under-represented based on annotation annotators. Discriminating differences between DmsD/ YnfI and its DmsABC and YnfEFGH were impossible beyond Gammaproteobacteria, since pairwise protein sequence identities between known *E. coli* components and suspected DMSO/selenate reductase components were equally low (8–28 %) even within many Gammaproteobacterial species. Due to low sequence identities between both reductases, the presence of two *dmsA* gene copies was used to identify potential *ynf* operons and the majority of duplicated *dmsA* were confidently identified in Gammaproteobacteria (as highlighted in Fig. 3).

### Operon analysis of NarJ subfamily loci had close associations between each member and its respective CISM system

Operon analysis of every gene located within a 10 ORF radius from each of the 324 NarJ subfamily sequence loci revealed that *narJ, narW*, and *dmsD* subfamily members had the strictest association to their CISM operons whereas *dmsD*, *torD* and *ycdY* loci were identified with diverse operons (Fig. 2). *narJ* loci demonstrated strict *cis* association (91.7 %) to its respective *narGHI* operon and all *narJ* loci were present between *narI* and *narH* genes in this operon (98.0 %) (Fig. 2). Other genes frequently identified with the *nar* operon were nitrate/ nitrite transporters of the major facilitator superfamily (MFS) commonly annotated as *narK* and *narU* (2A0108; PRK15034). At least one (57.7 %) or both of these transporter genes (21.9 %) were present in one *nar* operon per species suggesting that nitrate/nitrite transport is regulated by the *nar* operon. A parvulin-like peptidyl-prolyl isomerase (*ppiC*), known to participate in the folding and secretion of extracelluar and periplasmic proteins [49], was frequently identified (52.3 %) within *narGHI* operons of denitrifying Proteobacterial species (excluding Epsilonproteobacteria). The high frequency of *ppiC* occurrence suggests that this peptide modifying enzyme may participate in nitrate/nitrite reductase maturation or its activities may be regulated by the *narGHI* operon. Similar to *narJ*, all *narW* loci were strictly *cis*-associated with its respective nitrate reductase (*narZYV*) operon (100 %), located between *narV* and *narY,* and included the nitrate/nitrite transporter *narU* (98.0 %). This analysis indicates a close association between both nitrate reductase chaperones and their respective target reductase components (*narGHI/ narZYV*).

*Cis* associations between the DMSO reductase (*dmsABC*) and annotated *dmsD* loci were highly conserved (80 %) similar to *nar* operons (Fig. 2). Analysis of *dmsD* to *dmsABC* operon revealed that the genetic association between *dmsD/ynfI* and the selenate reductase operon (*ynfEFGH*) could only be was confidently phylogenetically identified in 7 Gammaproteobacterial species (Fig. 3). This indicated that *dmsD/ynfI* from all other phyla were linked to *dmsABC* operons. *Cis*-associated *dmsD* genes were frequently located after *dmsC* (*dmsABCD*) in the *dms* operon of Proteobacterial species (65 %) and before *dmsA* in Gram-positives (61 %) and Archaea (85 %) indicating that an organizational bias exists between Gram types. It is important to note that a few species possessed ‘*torD*’ annotated genes that were *cis*-associated with a *dmsABC* operon (7 % of all ‘*torD*’). These ‘*torD*’ genes likely represent mis-annotated *dmsD* members based on their phylogenetic association to other DmsD sequences and their lack of identifiable *torAC* (Fig. 3 and Additional file 6: Figure S2). The remaining *dmsD* loci (20 %) were associated with either the Fdh operon (*fdhGHI)* (10 %) or as part of a 2–3 ORF operon referred to as *nrfD*-*hybA*-*bisC* (10 %) (Fig. 2). This operon was composed of a formate-dependent nitrate reductase/ polysulfide reductase (*nrfD*), a hydrogenase 4Fe-4S ferredoxin-type component (*hybA*), and a putative anaerobic dehydrogenase/molybdopterin oxidoreductase (often annotated as a ‘biotin sulfoxide reductase *bisC* family member’ and/or *mopB*-like ‘molybdopterin-binding oxidoreductase-like domain containing’ protein). These *dmsD*- *fdhGHI* linked operons frequently possessed (60 %) a pair of upstream putative membrane proteins (DUF3305 and DUF3306) as well as a copy of the *hybA* gene suggesting these genes may be regulated by *dmsD*-*fdhGHI*. In some cases species with DmsD*fdh* or DmsD_*bisC*_ operons (40 % of Alphaproteobacteria, 75 % Actinobacteria, and 100 % Deferribacteres) possessed additional *dmsABC* and *dmsD* loci in *trans* to either operon*.* These *trans*-associated *dmsD* possessed extra annotated ‘*dmsD’* copies (1–5) and/or incomplete DMSO reductase operons frequently lacking the membrane anchor subunit *dmsC* making it difficult to determine precise associations between the multiple *dmsD* and *dmsABC* copies. These operons may reflect soluble forms of DMSO reductases similar to the single subunit DMSO reductase characterized in Alphaproteobacterial *Rhodobacter sphaeroides* since it only requires a Mo*bis*PGD cofactor [50]. Alternatively, these incomplete *dms* operons may be the remnants of partial lateral gene transfers, as transposon and integrase genes were noted within the 10 ORF gene radii of *dmsD* loci at 21 % frequency of occurrence (Fig. 3).

YcdY homologues were unevenly identified between two operon types, those that associated within the *ydcXY* (64 %) operon or to a newly identified 2 ORF operon (36 %) (Fig. 2). In some cases annotated *ycdY* were present as part of the *dmsABC* operon (~10 %) and phylogenetic and MSA analyses suggest that these sequences may actually represent *dmsD* rather than *ycdY* (Fig. 3 and Additional file 2: Figure S1)*.* All *ycdY* that were located as part of a *bona fide ycd* operon had a single copy of a polymerase/ histidinol alkaline phosphatase involved in swarming (*ycdX*) [35]. Almost all Proteobacterial *ycdY* were *cis*-linked to *ycdX* operons or lacked association with an operon but possessed at least one copy of *ycdX* in *trans*. Additional *ycd* operon genes were also infrequently identified and included an inner membrane transport protein of unknown function (*ycdZ*) and a 2-ketoacid/ bifunctional glyoxylate/hydroxypyruvate reductase (*ghrA/ ycdW*). Most Gammaproteobacterial *ycdY* were located within a *ycd* operon and many *ycdY* homologues were misannotated as ‘TorD’ according to phylogenetic analysis (Fig. 3). The remaining Gammaproteobacterial *ycdY* were identified as part of a 2 ORF operon composed of a Zn-dependent dehydrogenase/reductase (*hflC)* and lipoprotein (*mdaB*) (Fig. 2) indicating that Gammaproteobaterial *ycdY* may be linked to Zn-dependent enzyme maturation.

Annotated ‘*torD’* loci demonstrated the lowest association to its respective TMAO reductase *torAC* operon (19.5 %) by comparison to *narJ* and *dmsD*. Legitimate *torD* sequences based on phylogenetic association and *torAC* operon presence were only identified in Gram-negative species. The location of *torD* within the *torAC* operon (TorD_*torAC*_) was highly variable (either *cis* or *trans*) (Fig. 2). Upstream regulatory elements such as *torRS* and an upstream periplasmic TMAO reductase *torT* were often identified (48 %) within half of the surveyed *torACD* operons (48 %). The remaining annotated ‘*torD’* loci were identified within *fdhGHI* (TorD_*fdh*_) operons (28.0 %) and a 2–3 ORF *nrfD*-*hybA*/ferredoxin-*bisC* (TorD_*bisC-nrfD*_) operon (14.6 %). TorD_*fdh*_ operons also frequently possessed additional genes such as putative lipoproteins (DUF1439; PRK10598) and hypothetical ferredoxins (43 %). Within proteobacterial species lacking *torAC,* annotated ‘*torD*’ genes were primarily TorD_*fdh*_ operon associated, whereas TorD_*bisC-nrfD*_ was identified in Gram-positives, Chlorobia and Archaeal species. Finally, a small number of annotated *torD* genes (9 %) were adjacent to one or more of the *dmsABC* operon genes suggesting that these *torD* genes may either be mis-annotated *dmsD* or *torD* that were joined by transposition. Due to the low rate of transposition (1 %) identified within these operons it is more likely that these gene are mis-annotated.

### Phylogenetic analysis of NarJ subfamily proteins reveals a cladistic bias towards operon association

Phlyogenetic analysis of all 324 NarJ subfamily proteins was used to estimate and validate the relationships between NarJ, NarW, DmsD, TorD, and YcdY members. The NarJ subfamily BI phylogram shown in Fig. 3 resulted in 3 major resolvable clades. The first and most distal clade was composed entirely of NarJ sequences (1a–c) and the remaining 2–3 clades 2–3 were composed of polychotomous subclades enriched with either DmsD or TorD sequences.

The NarJ clade (1) demonstrated a subdivision of NarJ sequences into 5 main polychotomous subclades (1a–f) where each group was enriched with sequences from closely related phyla. A NarW subclade was noted in clade 1a (including *E. coli* NarW) and formed a proximal branch from Gammaproteobacterial NarJ sequences (including *E. coli* NarJ), reconfirming that NarW paralogously evolved from Proteobacterial NarJ [9]. Operon analyses of duplicate NarJ sequences (annotated as ‘NarJ2’) within the NarW subclade 1a revealed an association with *narZYV* operons. Actinobacterial and Firmicutes species with multiple NarJ sequences (clades 1b–c) demonstrated a close associated to NarJ sequences on adjacent branches, indicating that multiple NarJ sequences in Gram-positive were the result of recent gene duplications (clades 1b–c). Although polytomy within the NarJ clade prevented the identification of an originating NarJ sequence the majority of NarJ sequences appear to follow linear inheritance. However, the presence of transposon and integron sequences associated with NarJ clades 1e–f strongly support lateral gene transfer of *nar* operons in Archaeal and Epsilonproteobacterial species (Fig. 3).

DmsD and TorD (clades 2–3) demonstrated extensive polytomy that prevented the determination of a common origin between DmsD and TorD clades (Fig. 3). Closer examination of operon associations within clade 2a determined that all of these DmsD/TorD sequences were associated with *bisC-hybA-nrfD* operons. This association appears to be in agreement with the phylogenetic relationships of CISMs oxidoreductases [5], where polysulfide reductase/ formate-dependent nitrate reductase related systems were shown to be the distal relatives of the more recently evolved DMSO reductase systems. Many large DmsD/TorD clades were enriched with either DmsD (3a–f) or TorD (TorD_*torAC*_ and TorD_*fdh*_) as well as many smaller clades of annotated TorD/DmsD (3 g–l). DmsD and annotated ‘TorD’ sequences within clades 3a–f were linked to *dmsABC* operons, indicating all of these sequences represent *bona fide* DmsD family members. This association was supported by higher sequence identities of DmsD clade 3a–f sequences to *E. coli* DmsD than to *E. coli* TorD (Additional file 2: Figure S1). The organization of smaller DmsD/ TorD_*bisC*_ clades was only evident when their respective CISM operon associations were determined and based on sequence identity values determined from the MSA (Fig. 3 and Additional file 2: Figure S1).

The proteobacterial DmsD clade 3a–b also revealed that all Gammaproteobacterial YcdY (all originally annotated as ‘TorD’) have recently evolved from DmsD. Unexpectedly, the YcdY subclade itself was subdivided into 2 branches: a distal branch (YcdYa) composed of sequences that associated with *ycdX* operons that include *E. coli* YcdY (YcdYa) and second proximal branch (YcdYb) of annotated ‘TorD’ sequences that associated with a 2 ORF operon composed of a Zn-dependent dehydrogenase/reductase (*hflC)* and lipoprotein (*mdaB*) (Fig. 2). The presence of YcdY homologues within the Gammaproteobacterial DmsD subclade supports that both YcdYa and YcdYb homologues all originated from DmsD duplications that have both diverged to accommodate the maturation of Zn-cofactor dependent enzymes based on operon associations.

TorD clades showed a clear CISM operon association bias. TorD sequences with *torAC* operons were observed in a single clade termed ‘TorD_*torAC*_’ that was enriched with Alpha-, Beta- and Gammaproteobacterial TorD sequences (including *E. coli* TorD; b0998) providing strong support they all represented *bona fide* TorD members. An second clade had *torD* loci that corresponded to *fdhGHI* operons (TorD_*fdh*_), and was composed of annotated ‘TorD’ sequences from all Proteobacteria and Negativicutes phyla. This finding is intriguing, since all species with TorD_*fdh*_ sequences possessed *fdhD*/*fdhE* chaperones that were *trans* to the *fdh* operon and suggest that *torD*-*fdh* operon associations do not compensate for missing formate dehydrogenase chaperones. Despite their association TorD_*fdh*_ clade sequences does not preclude their functional involvement with a TMAO reductase. TorD_*fdh*_ sequences in (Gamma- Delta and Epsilon-proteobacteria) all possessed a detectable *trans torAC* operon to its *torD* locus (Additional file 5: Figure S5). Alpha- and Beta-proteobacterial ‘*torD*’ loci, all lacking detectable *torAC* operons, had TorD_*fdh*_ sequences that may be associated with periplasmic TMAO reductase (*torT*) and/or a periplasmic sulfite/TMAO/DMSO oxidase system (*yedYZ*) as they were detected in these strains (Additional file 5: Figure S5).

A final small TorD clade (TorD_*ttr*_) was composed of Firmicutes species with *torD* loci that associated with tetrathionate reductase system (*ttrABC*) operons (Fig. 3 and Additional file 5: Figure S5). The tetrathionate reductase chaperone (TtrD) protein was recently characterized as a maturase/chaperone for the molybdopterin-containing Archaeal *Archaeoglobus fulgidus* TtrABC tetrathionate reductase [51]. Annotated ‘*torD*’ sequences within the TorD_ttr_ clade may represent a mis-annotated branch of *ttrD*. The remaining TorD/DmsD sequences formed smaller subclades (3f–m) that were more difficult to discriminate *bona fide* DmsD from TorD. These clades were composed of a collection of annotated ‘TorD’, ‘DmsD’ and ‘NarJ’ members that were located in *bisC*-*hybA*-*nrfD* operons (Figs. 2 and 3). The majority of these TorD/DmsD_*bisC*_ clades could not be clearly explained by operon association alone. However, some of these clades demonstrated moderate correlations (*r* +0.71–0.74) to the presence or absence of other molybdopterin containing reductase enzymes such as polysulfide reductase (*psr*), thiosulfate reductase (*psh*), periplasmic nitrate reductases (*nap*/ *nir*), and xanthine dehydrogenase (*xdh*) operons suggesting their potential association to these reductases (Additional file 6: Figure S2 and Additional file 4: Figure S4).

### Non-synonymous to synonymous nucleotide substitution values identify NarJ subfamily members within specific phyla under purifying and neutral selection

**Fig. 4:**
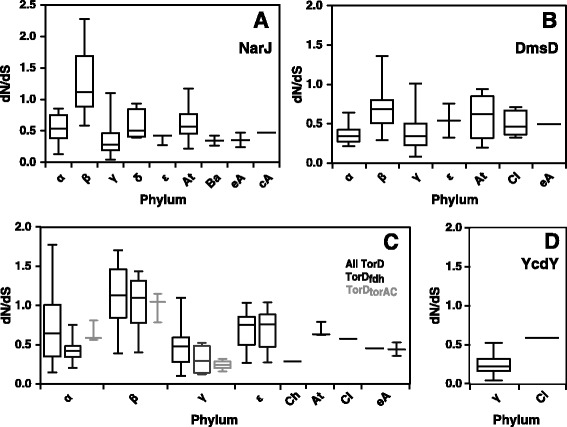
A summary of dN/dS rates for NarJ subfamily nucleotide sequences from Bacterial and Archaeal phyla. Each panel summarizes intra-phylum species dN/dS values for each NarJ subfamily member nucleotide codon alignment. Each boxplot indicates the maximum (top error bar), 75^th^ percentile, median (50^th^ percentile), 25^th^ percentile and minimum (bottom error bar) dN/dS value (y-axis). Phylum listed on the x-axis of each panel are abbreviated as follows: Alphaproteobacteria (α), Betaproteobacteria (b), Gammaproteobacteria (γ), and Deltaproteobacteria (δ), Epsilonproteobacteria (ε),Chlorobia (Ch), Actinobacteria (At), Bacilli (Ba), Clostridia (Cl), Euryarchaea (eA), and Crenarchaea (cA). Panel **a** shows dN/dS values for *narJ* codon aligned nucleotide sequences. Panel **b** provides dN/dS values for *dmsD* codon aligned nucleotide sequences. Panel **c** shows dN/dS values for all *torD* sequences (black), as well as dN/dS values from codon subalignments of *torD*_*fdh*_ (dark grey) and *torD*_*torAC*_ (light grey) subgroups. Panel **d** shows *ycdY* dN/dS values

The rate of non-synonymous to synonymous nucleotide substitutions (dN/dS) were estimated for intra-phylum pairwise comparisons of nucleotide sequences from each NarJ subfamily member to determine the type of selective pressures that may be influencing their evolution (Fig. 4). NarJ subfamily members with dN/dS < 1 indicate sequences influenced by purifying (negative) selection where some individual codons may be undergoing positive selection but not enough to overcome selection pressure that suppresses protein changes. Members with dN/dS = 1 suggest neutral selection where no clear selective pressure can be determined and dN/dS > 1 indicate members undergoing repeated positive selection that influence specific codon alterations [48, 52].

**Fig. 5:**
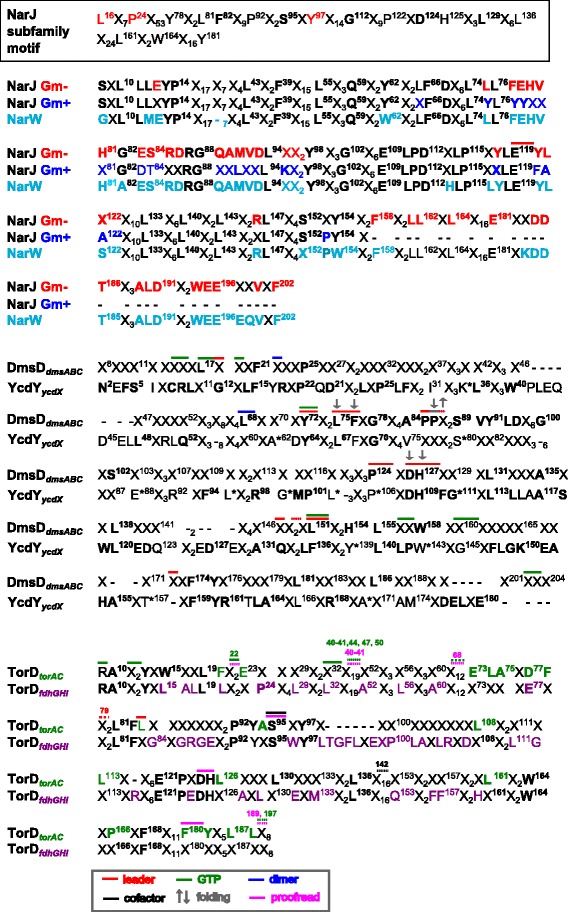
A summary of NarJ subfamily member motifs determined in this study. For all motifs, residues with 60-74 % (normal font) sequence identity and >75 % (bold font) sequence identity are shown. Dashes in each motif indicate unalignable or missing residue gaps within the alignment. The overall NarJ subfamily motif is provided in the top box and was determined from the overall alignment of 324 sequences (Additional file 5: Figure S5); NarJ subfamily motif numbering is according to aligned *E. coli* TorD positions; red residues indicate newly determined amino acids as determined from this study. Motifs determined from sub-alignments of 82 NarJ, 6 NarW, 74 DmsD, 13 YcdY and 97 TorD sequences are listed below the overall NarJ subfamily motif. In NarJ motifs, differences between Gram- negative (Gm-; red font) and Gram- positive (Gm+; blue font) sequences are highlighted in addition to overall NarJ motif (black font) and both motifs are numbered according to *E. coli* NarJ (b1226). The NarJ motif alignment included Gammaproteobacterial NarW motifs (light blue font) which are numbered according to *E. coli* NarJ. The DmsD motif represents sequences with loci found in *dmsABC* operons and is numbered according to its alignment to *E. coli* DmsD (b1591). The YcdY motifs for members linked to *ycdX* operons is aligned with DmsD and numbered according to *E. coli* YcdY (b1035). Starred YcdY motif residues indicate positions that differ in YcdYb sequence motifs (K37G/N, A62Q, S80A, E88V/I, L95R, G99N, L102E, Q123N, W143C, T157S/A, A170L/M/I). Both TorD motifs shown indicate members that associated with *torAC* operons (green font) and *fdh*GHI operons (purple font) in addition to the overall TorD motif (black font) that is numbered according to *E. coli* TorD (b0998). Residues with known experimental functional/ structural significance are highlighted with coloured bars or arrows (refer to panel legend for details)

According to mean dN/dS values for all NarJ subfamily members (NarJ 0.65, TorD 0.65, DmsD 0.57, and YcdY 0.24) all members were maintained under purifying selective pressure commonly observed for most genes [52]. Different rates of purifying selection were noted within phyla for each NarJ subfamily member indicating that negative selection varies within phyla and is expected to contribute to the extent of sequence variation/ divergence within subfamily members from these species. This is particularly noticeable when comparing dN/dS values between phylum with *narJ* and *torD*_*fdh*_ and *torD*_*torAC*_ members (Fig. 4a and c) and may reflect the motif differences identified for each these members (Fig. 5). Examination of intra-phylum dN/dS values for each subfamily member revealed different patterns and determined dN/dS values slightly above or equal to 1 within particular phyla for NarJ, DmsD and TorD (TorD_*fdh*_) members (Fig. 4) indicating that neutral selection is occurring within these phyla. Betaproteobacterial *narJ* and *torD* sequence comparisons resulted in dN/dS ≥ 1 at the median or 75^th^ percentile range indicating it was the only phyla evolving under neutral selection. To a lesser extent, dN/dS ≥ 1 were noted for 25 % intra-phylum species comparisons of *narJ*, *dmsD* and *torD* members indicating that particular pairwise comparisons between species, specifically between closely related species/ classes, also evolved under neutral selection. Comparison of NarJ subfamily members within closely-related species often results in very few substitutions, and consequently may reduce the accuracy in estimating dN/dS [53, 54]. Therefore, NarJ subfamily chaperones appear to be maintained by purifying selection (and neutral selection within members from specifc phyla) indicating that pressure suppressing overall protein sequence changes influences all subfamily members and reflects their role in the maturation of ancient CISM systems.

### Amino acid conservation and Sd codon rates identify unique motifs and conserved regions within each NarJ subfamily member

Multiple NarJ subfamily member sequence alignments and their corresponding nucleotide codon alignments were used to identify regions of residue conservation. It provided an opportunity to update NarJ subfamily motifs generated from Gammaproteobacterial sequences [7, 11], and the majority of these motif residues were still observed in this analysis (Fig. 5). This analysis identified additional NarJ subfamily motif residues L16, P24 and Y97 according to their alignment with *E.coli* NarJ and based on their high rate of occurrence (60–65 % identity) within MSA (Fig. 5 and Additional file 2: Figure S1). Some notable exceptions to the NarJ subfamily motif occurred at S95 in Gram-positive NarJ members (*E.coli* NarJ motif S84) and DmsD/ TorD_*bisC*_ (Fig. 5 and Additional file 2: Figure S1).

Examination of residue % identities and codon Sd rates within NarJ MSA revealed high residue/ codon conservation within the predicted α- helices 1, 4, 6, and 7 as well as moderate to poor conservation within the remaining C-terminal region (Additional file 5: Figure S5) indicating that the C-terminal region was undergoing the greatest amount of variation. To resolve the C-terminal region of NarJ, subalignments identified 2 distinct NarJ motifs patterns in the C-terminus (starting at *E. coli* NarJ 74–98) from sequences originating from Gram- negative versus and Gram- positive phyla (Fig. 5 and Additional file 2: Figure S1). In particular, Gram- negative NarJ sequences had a noticeable 22–27 residue extension (starting at *E.coli* NarJ D209) at the C-terminus. This may imply that the C-terminal extension present in Gram- negative NarJ sequences plays a specific role for these chaperones. The diversity and flexibility of this C-terminal extension prevented the generation of a 3D structure of *E. coli* NarJ by homology modeling until the 50 C-terminal residues were removed [55].

NarW sequence homology could only be confidently assigned to six Gammaproteobacterial sequences based on phylogeny (Fig. 3 and Additional file 4: Figure S4) and unsurprisingly its motif closely resembled the Gram-negative NarJ motif (Fig. 5 and Additional file 2: Figure S1). Despite the high overall similarity, some key differences in NarW alignments were noted, particularly at conserved residues within the N- and C-terminal ends of the protein. Also, some residues were missing in the N-terminus region of NarW; 7 residues between *E. coli* NarJ residues 37–44. The NarJ-NarW motif differences highlight important regions to consider examining in future experiments.

DmsD_*dmsABC*_ MSA identified additional residues positions in the updated motif that may help discriminate these chaperones from other NarJ subfamily members (Fig. 5 and Additional file 5: Figure S5). Analysis of individual residue % identities and codon Sd rates across DmsD_*dmsABC*_ sequences determined that regions of high residue/ codon conservation occurred within predicted C-terminal α- helices (5–12) and within loops/turns (2, 5, 6, 7) and poor (10–40 %) conservation of most positions within the N-terminus region (Additional file 5: Figure S5). This indicates that greater selection pressures act upon the C-terminal half of DmsD proteins. Most experimentally determined residues involved in leader peptide interactions [56, 57] and folding stability [58, 59] occurred in the highly conserved region of DmsD emphasizing its importance (Fig. 5 and Additional file 5: Figure S5). Residues predicted to be involved in GTP-binding did not frequently coincide with highly conserved DmsD residues [60] possibly suggesting that these interactions are more flexible or possibly linked to nearby unidentified conserved residues.

TorD MSA composed of TorD_*torAC*_ and TorD_*fdh*_ sequences only demonstrated poor to moderate (20–60 %) conservation the within the N-terminal region and greater conservation (>75 %) in central and C-terminus loops (4,6,8) as well as in α-helices (5, 7, 8, 10) (Additional file 5: Figure S5). Separate TorD_*torAC*_ and TorD_*fdh*_ subalignments revealed distinct motifs between both operon associated chaperones (Fig. 5). Residue conservation between in TorD_*torAC*_ and TorD_*fdh*_ sequences were shown to differ considerably around C-terminal α-helix 10 based on alignments to the current X-ray crystal secondary structure (1N1C [61]) (Additional file 5: Figure S5). Similar to DmsD, experimentally determined residues involved in GTP-binding did not coincide with conserved residues [33, 34, 60] suggesting that GTP-binding sites in TorD are less essential or prone to greater variation within the protein. A third of the characterized TorD residues shown to interact with the leader signal peptide, proofreading, and co-factor insertion [30, 32–34] occurred at conserved positions within the central region of the protein (α-helices 6 and 8). Coincidentally, the region between helices 6 and 7 of the *Shewanella massilia* TorD structure is a hinge that connects a unique domain-swapped homodimeric structure of TorD [61]. This hinge was demonstrated to be important for *E. coli* TorD recognition of the leader peptide of the Mo*bis*PGD-containing subunit of TMAO reductase [32]. All remaining leader-binding, proofreading and co-factor insertion linked residues aligned to moderately (40–60 %) conserved regions (Fig. 5 and Additional file 5: Figure S5) suggesting that these sites may be less crucial in other species/ phyla based on the high extent of TorD sequence variation.

YcdY MSA revealed a motif with a closer resemblance to DmsD rather than TorD (Fig. 5 and Additional file 5: Figure S5). Lower sequence conservation (20–60 %) was noted for predicted α-helices 2–3 and for central α-helices 6–7 and greater residue conservation was detected in the C-terminal region of YcdY (Fig. 5 and Additional file 5: Figure S5). Similar to phylogenetic analyses, differences between YcdY_*ycdX*_ and YcdY_*hflC-mdaB*_ motifs were noted and may identify key residues/regions that can guide further subfamily member structure-function analyses.

## Discussion

**Fig. 6:**
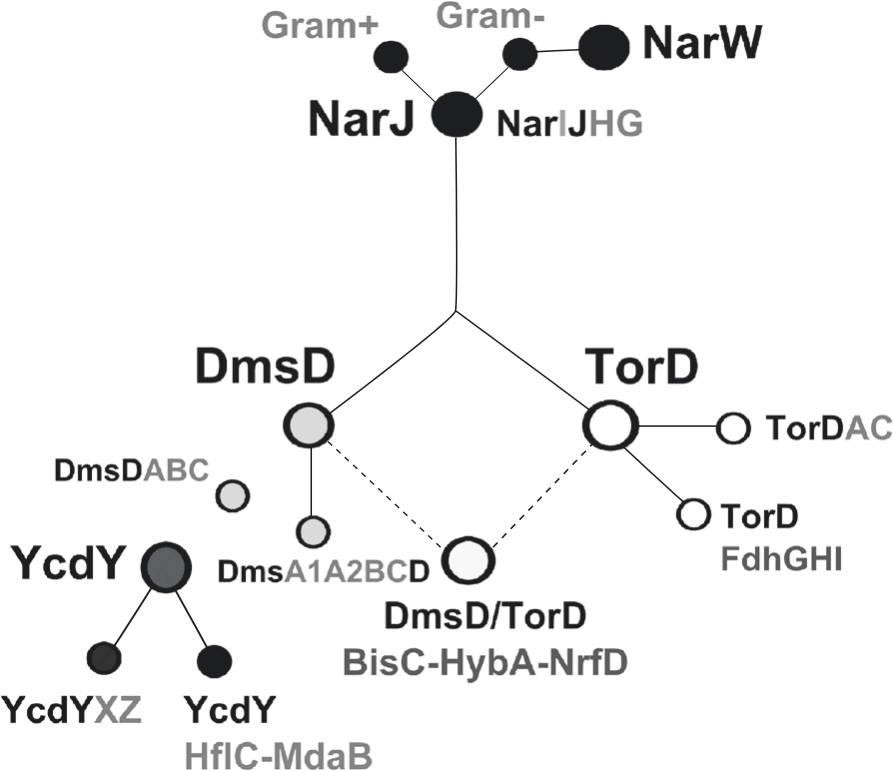
A summary dendrogram of NarJ subfamily family member relationships determined in this study. Circle nodes in the dendrogram identify specific NarJ subfamily members, NarJ/ NarW (black filled), DmsD (dark grey filled), YcdY (light grey filled) and TorD (white filled). Lines represent relationships only (no distances are provided) and connect NarJ subfamily members based on phylogenetic (Fig. 3) and protein identities determined from the NarJ subfamily MSA (Additional file 5: Figure S5)

Bioinformatics analyses of taxonomically diverse NarJ subfamily members NarJ, NarW, DmsD, TorD and YcdY, have revealed that a close association exists between each chaperone and a specific CISM system/ operon. This study provides further evidence that supports the system specificity of each NarJ subfamily member during CISM maturation and a summary of these findings are provided in Fig. 6. NarJ members demonstrated the strictest conservation when compared to all NarJ subfamily members, regarding its sequence motif as well as its association to *narGHI* operons. The close association of *narGHJI* across all phyla may be an excellent example of selfish operon theory [62], since many respiratory enzymes represent some of the most ancient genes in Bacteria and Archaea [5]. NarJ sequence motifs from Gram-positive and Gram-negative species also highlight potential differences in function or in the ability to differentiate specific nitrate reductases. Gram-negative NarJ members serve as progenitors of Gammaproteobacterial NarW paralogues. This contrasts Gram- positive NarJ members that appear to lack NarW paralogs but have closely related NarJ duplications based on their phylogenetic associations (Figs. 3 and 6). Phylogenetic analysis using different NarJ subfamily member outgroups supported that NarJ serves as the progenitor of subfamily members DmsD and TorD and is in agreement with a recent phylogenetic study of CISMs that demonstrated NarGHI homologues were distantly related to DmsABC systems [5].

The evolutionary analysis of DmsD and TorD members together, confirmed that YcdY members were paralogues of Gram-negative DmsD members, despite their frequent ‘TorD’ annotation. Two distinct YcdY subclades were identified in Gammaproteobacteria species, those possessing YcdY associated to *ycdX* operons [35] and a more recent subclade (YcdYb) that linked to a *hflC* and *mdaB* operon. *HflC* is an inner membrane component of the HflK-HflC complex that includes HflB, an integral membrane ATP-dependent zinc metallopeptidase, and functions as a regulator of FtsH protease [63, 64]. Although experiments examining the folding translocation of HflKC determined that it required a Sec- dependent pathway [64], YcdY may play an important role in the co-factor insertion of other Zn-dependent complexes as experimentally demonstrated for YcdX [35]. The lipoprotein *mdaB* functions as a quinone reductase, it is dependent on NADPH and is active with quinone derivatives using ferricyanide as electron acceptors [65]. The presence of *mdaB* may suggest that YcdY participates in quinone reductase maturation. Therefore, examination of YcdY/TorD operons in this study has identified other potential enzyme substrates that may involve YcdY-mediated maturation and merit future consideration.

Although *dmsD-dmsABC* conservation and genetic organization was high in this study, similar to the *narGHJI* operons, some *dmsD* loci (20 %) were associated to other operons. This suggests that *dmsD-dmsABC* associations were subjected to greater genetic re-arrangement/re-organizations than NarJ. Many phyla possessed additional copies of *dmsD* in *trans* from additional copies of complete and incomplete *dmsABC,* suggesting that DmsD and DmsABC enzymes are frequent targets of gene/ operon duplication. It is uncertain why DmsD and its cognate CISM have undergone such genetic variation by comparison to NarJ, but understanding the CISM distribution, inheritance and/or the type of respiration performed by their cognate enzymes may provide some explanation. Since evidence of lateral gene transfer was identified for DmsD as well as NarJ and TorD members (18–25 %), the close association between DmsD chaperone and its DMSO reductase still supports the currently proposed selfish operon theory [66], making this subfamily and CISM/ Zn-dependent enzyme an excellent operon family to explore further. The close linkage of *dmsD* to *dmsABC* operons have not only aided the determination of *bona fide* DmsD members but also indicated that many organisms may benefit from having additional *dmsD* copies regulated by other CISM operons as DmsD_*fdh*_ and DmsD_*bisC*_, especially in species with incomplete *dmsABC* operons. This provides further support for DmsD’s role as a maturation hub in the folding of other CISM systems. The high degree of sequence variation within the N-terminus of DmsD protein alignments may reflect changes in specificity between different substrates like DMSO reductase alpha subunit component DmsA [25] or the selenate reductase alpha components YnfE and YnfF [26] and further studies of DmsD-linked selenate reduction in species beyond Gammaproteobacteria may help clarify *ynfI* and *dmsD* homology.

TorD subfamily members demonstrated the most variation regarding its genetic association to its respective *torAC* operon. Out of the 130 species surveyed with identifiable NarJ subfamily members, only 1/3 of species with one or more annotated ‘*torD’* homologues actually possessed a copy of *torA* and/or *torC* genes. This indicated that the remaining 2/3 of *torD* sequences were mis-annotated or participate in the maturation of other CISM systems such as TorD_fdh_ and TorD_*bisC*_. Since TorD_*torAC*_ associations only appear to be identified in Proteobacteria, more ancient versions of *torD* may be linked to the maturation other CISM systems that utilise similar substrates. This may explain why 1/3 of all ‘*torD’* loci were associated with *fdhGHI* operons. Since FdhD/ E were almost always detected (97 %) in *trans* to all TorD_*fdh*_ species it is unlikely that TorD functionally compenstates for FdhD/E chaperones. However, almost all species with TorD_*fdh*_ had a high correlation (*r* + 0.88) to *trans* copies of periplasmic molybdoprotein reductase complex (*yedYZ*) operons, known to contain a TAT signal peptide [67]. Associations between *torD* and *fdhGHI* operons may be explained by the evolutionary origins of CISMs themselves. Phylogenetic analysis of Prokaryotic molybdopterin-containing respiratory enzymes suggested that FdhGHI were more distantly related to NarGHI, and then evolved to form DmsABC [5]. If this is correct, TorD associations to FdhGHI may reflect an ancient operon divergence between FdhD/E and TorDAC to accommodate the emergence of YedZY. The clearest evidence supporting *torD* divergence was highlighted by distinct motif differences between TorD_*fdh*_ and TorD_*torAC*_. These motif differences strongly suggest that the genetic association to each operon was evolutionarily directed towards different systems and regulation.

The final 1/3 of TorD_*bisC*_ and 20 % of DmsD_*bisC*_ were difficult to discern from each other due to the number of small monophyletic subclades within the DmsD/ TorD clade (2b) (Figs. 3 and 6). One explanation is that these *bisC-hybA-nrfD* associated members have diverged with in these species to accommodate CISMs with specific substrates, as observed for the tetrathionate reductase linked TorD_*ttr*_ subclade (Fig. 3). DmsD/TorD_*bisC*_ may have diverged to assist in the maturation of additional CISMs such as polysulfide reductases (Psr), thiosulfate reductases (Psh), and xanthine dehydrogenases (Xdh), were present (correlated at *r* +0.71 to +0.78) in DmsD/TorD_*bisC*_ subclades (Additional file 4: Figure S4). These members may participate in the maturation of *bisC* and *nrfD* complexes. BisC is a CISM enzyme responsible for biotin-d-sulfoxide and methionine-S-sulfoxide reduction and to a lesser extent detoxification of N-hydroxylated bases. It plays a role in biotin scavenging and assimilation of oxidized methionines [68–70]. Since BisC is known to be dependent on an as-yet unidentified chaperone for its maturation [71], DmsD/TorD_*bisC*_ sequences are worthy candidates for future consideration. Additionally, formate-dependent nitrate reductase/polysulfide reductase NrfD is known to be an inner membrane protein which has demonstrated both naphthtoquinol oxidase and proton pump activities [72, 73]. Altogether, diverse genetic associations observed within DmsD/TorD_*bisC*_ subclades have likely contributed to their low overall conservation within sequence sub-alignments. The relationship of DmsD/TorD_*bisC*_ members will remain elusive until more is known about these *bisC-hybA-nrfD* operons.

## Conclusions

The evolution and diversity of NarJ subfamily members NarJ, DmsD, TorD, and YcdY all appear to be highly influenced by their genetic association to CISM enzyme genes. Selection pressures acting on each subfamily member differs considerably from N- to C- terminus confirming that each family member is diverging in sequence to conform to its cognate respiratory enzyme. NarJ subfamily members appear to be diverging into paralogous subfamilies that specifically target other CISM, such as NarJ and NarW, and possibly TorD (TorD_*torAC*_ and TorD_*fdh*_). DmsD was demonstrated to be the origin of chaperones that require an alternative cofactor like Zn as demonstrated for YcdY_*ycdX*_/YcdY_*hflC-mdaB*_ indicating that DmsD members also serve as a chaperone progenitor. The significance of these findings indicate that NarJ subfamily members are highly diverse and tailored to accommodate a variety of respiratory and non-respiratory co-factor requiring systems making them fascinating proteins for further analysis to expand the current maturation/chaperone proteome.

## Additional files

Additional file 1: Table S1. A list of all species with NarJ subfamily members examined in this study and their respective gene locus tags and protein accession numbers. Additional file 2: Figure S1. An unedited multiple sequence alignment (MSA) of 324 NarJ subfamily proteins examined in this study. The MSA shown represents a consensus between Cobalt [38] and Praline [39] MSA programs. All protein sequences shown in the alignment are unedited. Positions with >80 % gaps were removed from this alignment to perform phylogenetic analysis and Sd/Nd analysis to avoid excessive gap penalties and prevent analysis errors caused by gaps. Each sequence is labelled according to its species, NarJ subfamily annotation (according to tBLASTn e-values < 1.0 × 10^−4^) and its respective Genbank/ NCBI Gene locus tag. Additional file 3: Figure S3. Rooted BI and NJ phylograms of NarJ subfamily sequences generated using Crenarchaeal NarJ, DmsD or TorD sequences as outgroups. A total of 6 rooted phylograms of 324 protein sequences from 130 different Prokaryotic species is shown after NJ and BI analyses using *Vulcanisaeta distributa* NarJ, *Thermoproteus tenax* TorD or *Vulcanisaeta distributa* DmsD sequences as outgroups. For BI phylograms, 10 million generations were performed and posterior probability values (0–1) at each node of the dendrogram are shown to provide branch confidence. Rooted NJ phlyograms of 324 protein sequences were determined from a consensus of 1000 bootstrapped replicate trees and confidence values (as a total of 1000) are indicated beside each node in the dendrogram. In all panels the genus and species and NarJ subfamily annotation are listed on the right branches and coloured according to NarJ subfamily annotation (NarJ blue; DmsD red; TorD green; YcdY yellow). Additional file 4: Figure S4. The rooted NJ phylogram of 324 NarJ subfamily proteins and heatmap of their respective CISM operon associations. The *Vulcanisaeta distributa* NarJ rooted NJ phlyogram of 324 protein sequences was determined from a consensus of 1000 bootstrapped replicates and confidence values (as a total of 1000) are indicated beside each node in the dendrogram. Coloured heatmaps on the right-hand side of REMP Genus species labels indicate the operon association of the subfamily member, the presence of one or more CISM operons (*narGHI*, *dmsABC*, *torAC* and *ycdX*), and other CISMs indentified within each species genome (refer to legend in panel and CISM operon definitions provided in Additional file 6: Figure S2). Additional file 5: Figure S5. A summary of NarJ subfamily amino acid percentage identities and Sd codon values determined from MSA and syn-scanning analysis. TorD residue % identity and Sd codon values for sequences that were genetically located within *fdh* operons (E) or *torAC* operons (F) are shown. Both charts show the residue identity (as a decimal value 0-1; black lines) and synonymous nucleotide rate (Sd) per codon (grey lines) that correspond to each numbered amino acid/ codon position (x-axis) in the overall NarJ subfamily alignment (in black font) or to the specified *E. coli* subfamily member (in grey font). Predicted secondary structures (α-helices; grey rectangles, and turns/loops; lines) are shown as a cartoon line diagram and α-helical secondary structure shown as cylinders correspond to the *E. coli* DmsD (panel B; 3EFP, 3CW0, 3U41 [50, 57, 60]) or *S. massila* TorD (panel A; 1N1C [61]) crystal structures below both residues alignments. Residues known to have experimental and/or predicted involvement in TAT leader/signal sequence binding (l), leader proofreading (p), co-factor insertion (c), GTP binding (g), folding stability (f), dimerization (d) and REMP motif (m) are provided below structural information. Additional file 6: Figure S2. A summary heatmap of NarJ subfamily operons and their respective CISM complex genes detected in all 130 Prokaryotic species genomes. Genus and species are listed on the y-axis and grouped alphabetically by phylum (according to the color bar on the left hand y-axis). Each column indicates the presence (coloured) and absence (white/uncoloured) of one or more of the genes listed on the upper x-axis of the dendrogram. The top x-axis indicates the subfamily member-CISM operon associations (left-hand columns) and other CISM genes/ operons identified from genome searches of each species. NarJ subfamily members termed “other” refer to sequences annotated as ‘nitrate reductase delta’ and/or ‘REMP’. Definitions for abbreviated CISM operons are as follows: *bisC-nrfD*; biotin sulfoxide reductase (-*hybA)* -polysulfide reductase; *fdhGHI*; formate dehydrogenase, *napABCHG*; periplasmic nitrate reductase, *nirBD*; cytosolic nitrate reductase, *nuoGHI*; NADH:ubiquinone oxidoreductase I, *phsABC*; polysulfide reductase; *sox*; sulfoxide reductase (includes methionine sulfoxide reductases and sulfite oxidases), *ttrABC*; tetrathionate reductase, *ydeP*; periplasmic acid resistance protein/ formate dehydrogenase homologue, *yedZY*; putative Mo*bis*PD oxidoreductase.

## Abbreviations

CISM: Complex iron-sulfur molybdoenzyme
DMSO: Dimethylsulfoxide
Dms: Dimethylsulfoxide reductase
Fdh: Formate dehydrogenase
Nar: Nitrate reductase
Mo*bis*PGD: Molybdenum-*bis* pyranopterin guanine dinucleotide)
MSA: Multiple sequence alignment
dN/dS: Non-synonymous to synonymous substitution rate
REMP: Redox enzyme maturation protein
TMAO: Trimethylamine-N-oxide
Tor: Trimethylamine-N-oxide reductase
