# Supplementary material for: NarJ subfamily system specific chaperone diversity and evolution is directed by respiratory enzyme associations
Source: BMC Evol Biol. 2015 Jun 12;15:110. doi: 10.1186/s12862-015-0412-3 (PMC4464133; doi:10.1186/s12862-015-0412-3)
Supplement: Additional file 1: Table S1. — A list of all species with NarJ subfamily members examined in this study and their respective gene locus tags and protein accession numbers. [file 12862_2015_412_MOESM1_ESM.docx]

**Additional file 1**

**Table S1.** A list of all species with NarJ subfamily members examined in this study and their respective gene locus tags and protein accession numbers.

| **Phylum** | **Class** | **Genus and species** | **NarJ sub-family** | **Gene locus tag** | **Protein accession number** |
| --- | --- | --- | --- | --- | --- |
| **Gram-positives** | | | | | |
| Actinobacteria | Actinobacteria | *Acidothermus cellulolyticus* | NarJ | Acel_0507 | ABK52280 |
| Actinobacteria | Actinobacteria | *Actinoplanes missouriensis 431* | NarJ | AMIS_40140 | BAL89233 |
| Actinobacteria | Actinobacteria | *Actinosynnema mirum DSM 43827* | NarJ | Amir_2038 | ACU35984 |
| Actinobacteria | Actinobacteria | *Amycolicicoccus subflavus DQS3-9A1* | NarJ | AS9A_2094 | AEF40543 |
| Actinobacteria | Actinobacteria | *Arthrobacter phenanthrenivorans Sphe3* | NarJ | Asphe3_35220 | ADX74623 |
| Actinobacteria | Actinobacteria | *Beutenbergia cavernae DSM 12333* | NarJ | Bcav_2805 | ACQ81050 |
| Actinobacteria | Actinobacteria | *Corynebacterium glutamicum ATCC 13032* | NarJ | cg1342 | CAF19891 |
| Actinobacteria | Actinobacteria | *Cryptobacterium curtum* | DmsD | Ccur_00520 | YP_003150467 |
| Actinobacteria | Actinobacteria | *Eggerthella lenta DSM 2243* | YcdY | Elen_0465 | YP_003180840 |
| Actinobacteria | Actinobacteria | *Eggerthella lenta DSM 2243* | DmsD | Elen_0425 | YP_003180803 |
| Actinobacteria | Actinobacteria | *Eggerthella lenta DSM 2243* | TorD | Elen_3035 | YP_003183367 |
| Actinobacteria | Actinobacteria | *Eggerthella lenta DSM 2243* | TorD2 | Elen_2380 | ACV56338 |
| Actinobacteria | Actinobacteria | *Eggerthella lenta DSM 2243* | TorD3 | Elen_2368 | ACV56326 |
| Actinobacteria | Actinobacteria | *Eggerthella lenta DSM 2243* | TorD4 | Elen_1192 | ACV55162 |
| Actinobacteria | Actinobacteria | *Eggerthella lenta DSM 2243* | REMP1 | Elen_0493 | ACV54478 |
| Actinobacteria | Actinobacteria | *Eggerthella lenta DSM 2243* | REMP2 | Elen_0477 | ACV54462 |
| Actinobacteria | Actinobacteria | *Eggerthella lenta DSM 2243* | TorD5 | Elen_0115 | ACV54109 |
| Actinobacteria | Actinobacteria | *Eggerthella lenta DSM 2243* | REMP3 | Elen_2920 | ACV56865 |
| Actinobacteria | Actinobacteria | *Eggerthella lenta DSM 2243* | TorD6 | Elen_2765 | ACV56712 |
| Actinobacteria | Actinobacteria | *Eggerthella lenta DSM 2243* | TorD7 | Elen_2402 | ACV56360 |
| Actinobacteria | Actinobacteria | *Eggerthella lenta DSM 2243* | REMP4 | Elen_1590 | ACV55558 |
| Actinobacteria | Actinobacteria | *Eggerthella lenta DSM 2243* | TorD8 | Elen_0613 | ACV54598 |
| Actinobacteria | Actinobacteria | *Eggerthella lenta DSM 2243* | REMP5 | Elen_3049 | ACV56992 |
| Actinobacteria | Actinobacteria | *Gordonia bronchialis DSM 43247* | NarJ | Gbro_3701 | ACY22885 |
| Actinobacteria | Actinobacteria | *Gordonibacter pamelaeae 7-10-1-b* | YcdY/ REMP | GPA_10020 | YP_007802133 |
| Actinobacteria | Actinobacteria | *Gordonibacter pamelaeae 7-10-1-b* | DmsD | GPA_11560 | YP_007802234 |
| Actinobacteria | Actinobacteria | *Gordonibacter pamelaeae 7-10-1-b* | REMP1/HyaE | GPA_25500 | BL04663 |
| Actinobacteria | Actinobacteria | *Gordonibacter pamelaeae 7-10-1-b* | REMP2 | GPA_32310 | CBL05043 |
| Actinobacteria | Actinobacteria | *Gordonibacter pamelaeae 7-10-1-b* | REMP3 | GPA_27010 | CBL04750 |
|  |  |  |  |  |  |
|  |  |  |  |  |  |
| **Phylum** | **Class** | **Genus and species** | **NarJ sub-family** | **Gene locus tag** | **Protein accession number** |
| Actinobacteria | Actinobacteria | *Gordonibacter pamelaeae 7-10-1-b* | REMP4/DmsD | GPA_25600 | CBL04668 |
| Actinobacteria | Actinobacteria | *Gordonibacter pamelaeae 7-10-1-b* | REMP5 | GPA_21770 | CBL04470 |
| Actinobacteria | Actinobacteria | *Gordonibacter pamelaeae 7-10-1-b* | REMP6/DmsD | GPA_13980 | CBL04017 |
| Actinobacteria | Actinobacteria | *Mobiluncus curtisii ATCC 43063* | NarJ | HMPREF0573_11448 | ADI67767 |
| Actinobacteria | Actinobacteria | *Mobiluncus curtisii ATCC 43063* | DmsD | HMPREF0573_11020 | ADI67339 |
| Actinobacteria | Actinobacteria | *Mycobacterium smegmatis str. MC2 155* | NarJ | MSMEG_5138 | ABK69889 |
| Actinobacteria | Actinobacteria | *Propionibacterium acnes SK137* | NarJ | HMPREF0675_3555 | YP_003580735 |
| Actinobacteria | Actinobacteria | *Propionibacterium acnes SK137* | TorD | HMPREF0675_3544 | ADD99751 |
| Actinobacteria | Actinobacteria | *Salinispora arenicola CNS-205* | NarJ1 | Sare_3140 | ABV98948 |
| Actinobacteria | Actinobacteria | *Salinispora arenicola CNS-205* | NarJ2 | Sare_0194 | ABV96129 |
| Actinobacteria | Actinobacteria | *Slackia heliotrinireducens DSM 20476* | DmsD | Shel_04330 | YP_003142843 |
| Actinobacteria | Actinobacteria | *Slackia heliotrinireducens DSM 20476* | TorD | Shel_20880 | YP_003144448 |
| Actinobacteria | Actinobacteria | *Slackia heliotrinireducens DSM 20476* | TorD1 | Shel_02390 | ACV21307 |
| Actinobacteria | Actinobacteria | *Slackia heliotrinireducens DSM 20476* | REMP3 | Shel_00630 | ACV21137 |
| Actinobacteria | Actinobacteria | *Slackia heliotrinireducens DSM 20476* | REMP2 | Shel_07920 | ACV21848 |
| Actinobacteria | Actinobacteria | *Slackia heliotrinireducens DSM 20476* | REMP1 | Shel_07890 | ACV21845 |
| Actinobacteria | Actinobacteria | *Slackia heliotrinireducens DSM 20476* | REMP4 | Shel_16920 | ACV22711 |
| Actinobacteria | Actinobacteria | *Slackia heliotrinireducens DSM 20476* | DmsD1 | Shel_06850 | ACV21744 |
| Actinobacteria | Actinobacteria | *Slackia heliotrinireducens DSM 20476* | TorD2 | Shel_07330 | ACV21791 |
| Actinobacteria | Actinobacteria | *Slackia heliotrinireducens DSM 20476* | TorD3 | Shel_27270 | ACV23725 |
| Actinobacteria | Actinobacteria | *Slackia heliotrinireducens DSM 20476* | TorD4 | Shel_27590 | ACV23756 |
| Actinobacteria | Actinobacteria | *Slackia heliotrinireducens DSM 20476* | TorD5 | Shel_05620 | ACV21621 |
| Actinobacteria | Actinobacteria | *Slackia heliotrinireducens DSM 20476* | DmsD2 | Shel_06160 | ACV21675 |
| Actinobacteria | Actinobacteria | *Slackia heliotrinireducens DSM 20476* | TorD6 | Shel_17900 | ACV22809 |
| Actinobacteria | Actinobacteria | *Slackia heliotrinireducens DSM 20476* | TorD7 | Shel_18510 | ACV22869 |
|  |  |  |  |  |  |
| **Phylum** | **Class** | **Genus and species** | **NarJ sub-family** | **Gene locus tag** | **Protein accession number** |
| Actinobacteria | Actinobacteria | *Slackia heliotrinireducens DSM 20476* | REMP5 | Shel_22270 | ACV23237 |
| Actinobacteria | Actinobacteria | *Slackia heliotrinireducens DSM 20476* | REMP6 | Shel_22800 | ACV23290 |
| Actinobacteria | Actinobacteria | *Slackia heliotrinireducens DSM 20476* | TorD8 | Shel_27400 | ACV23738 |
| Actinobacteria | Actinobacteria | *Streptomyces coelicolor A3(2)* | NarJ | SCO6533 | NP_630614 |
| Actinobacteria | Actinobacteria | *Streptomyces coelicolor A3(2)* | NarJ3 | SCO4949 | CAD30935 |
| Actinobacteria | Actinobacteria | *Streptomyces coelicolor A3(2)* | NarJ2 | SCO0218 | CAB53442 |
| Firmicutes | Bacilli | *Bacillus atrophaeus 1942* | NarJ | BATR1942_16590 | ADP34237 |
| Firmicutes | Bacilli | *Bacillus selenitireducens MLS10* | NarJ | Bsel_1294 | YP_003699372 |
| Firmicutes | Bacilli | *Bacillus selenitireducens MLS10* | TorD | Bsel_2605 | YP_003700670 |
| Firmicutes | Bacilli | *Bacillus selenitireducens MLS10* | DmsD2 | Bsel_3102 | ADI00584 |
| Firmicutes | Bacilli | *Bacillus selenitireducens MLS10* | DmsD1 | Bsel_1860 | ADH99365 |
| Firmicutes | Bacilli | *Bacillus selenitireducens MLS10* | TorD2 | Bsel_1479 | ADH9899 |
| Firmicutes | Bacilli | *Bacillus selenitireducens MLS10* | TorD3 | Bsel_0564 | ADH98100 |
| Firmicutes | Bacilli | *Geobacillus thermodenitrificans NG80-2* | NarJ1 | GTNG_0655 | YP_001124780 |
| Firmicutes | Bacilli | *Geobacillus thermodenitrificans NG80-2* | NarJ2 | GTNG_1714 | YP_001125823 |
| Firmicutes | Bacilli | *Macrococcus caseolyticus JCSC5402* | NarJ | MCCL_0138 | BAH16845 |
| Firmicutes | Bacilli | *Paenibacillus polymyxa M1* | NarJ | PPM_3622 | CCI70431 |
| Firmicutes | Bacilli | *Staphylococcus epidermidis ATCC 12228* | NarJ | SE1973 | AAO05614 |
| Firmicutes | Lactobacilli | *Lactobacillus plantarum JDM1* | NarJ | JDM1_1255 | CCC78827 |
| Firmicutes | Clostridia | *Alkaliphilus metalliredigens QYMF* | DmsD | Amet_1329 | YP_001319190 |
| Firmicutes | Clostridia | *Alkaliphilus metalliredigens QYMF* | TorD | Amet_0992 | YP_001318868 |
| Firmicutes | Clostridia | *Clostridium pasteurianum BC1* | DmsD | Clopa_0515 | YP_007939243 |
| Firmicutes | Clostridia | *Clostridium pasteurianum BC1* | YcdY | Clopa_4250 | AGK98973 |
| Firmicutes | Clostridia | *Desulfitobacterium dehalogenans ATCC 51507* | DmsD | Desde_2043 | YP_006430220 |
| Firmicutes | Clostridia | *Desulfitobacterium dehalogenans ATCC 51507* | YcdY | Desde_0500 | YP_006428754 |
| Firmicutes | Clostridia | *Desulfitobacterium dehalogenans ATCC 51507* | TorD | Desde_1715 | YP_006429907 |
| Firmicutes | Clostridia | *Desulfitobacterium dehalogenans ATCC 51507* | REMP | Desde_1718 | AFM00117 |
| Firmicutes | Clostridia | *Desulfitobacterium dehalogenans ATCC 51507* | DmsD2 | Desde_1889 | AFM00281 |
| Firmicutes | Clostridia | *Desulfitobacterium dehalogenans ATCC 51507* | DmsD3 | Desde_4013 | AFM02275 |
| Firmicutes | Clostridia | *Desulfitobacterium dehalogenans ATCC 51507* | DmsD4 | Desde_1205 | AFL99634 |
|  |  |  |  |  |  |
| **Phylum** | **Class** | **Genus and species** | **NarJ sub-family** | **Gene locus tag** | **Protein accession number** |
| Firmicutes | Clostridia | *Desulfitobacterium dehalogenans ATCC 51507* | REMP2 | Desde_1876 | AFM00268 |
| Firmicutes | Clostridia | *Desulfitobacterium dehalogenans ATCC 51507* | REMP3 | Desde_1855 | AFM00248 |
| Firmicutes | Clostridia | *Desulfitobacterium dehalogenans ATCC 51507* | REMP4 | Desde_1413 | AFL99836 |
| Firmicutes | Clostridia | *Desulfitobacterium dehalogenans ATCC 51507* | DmsD5 | Desde_3925 | AFM02192 |
| Firmicutes | Clostridia | *Desulfitobacterium dehalogenans ATCC 51507* | REMP5 | Desde_3584 | AFM01859 |
| Firmicutes | Clostridia | *Desulfitobacterium dehalogenans ATCC 51507* | REMP6 | Desde_0473 | AFL98937 |
| Firmicutes | Clostridia | *Desulfitobacterium dehalogenans ATCC 51507* | DmsD6 | Desde_0305 | AFL98775 |
| Firmicutes | Clostridia | *Desulfitobacterium dehalogenans ATCC 51507* | REMP7 | Desde_2002 | AFM00389 |
| Firmicutes | Clostridia | *Desulfitobacterium dehalogenans ATCC 51507* | REMP8 | Desde_4088 | AFM02349 |
| Firmicutes | Clostridia | *Desulfosporosinus orientis DSM 765* | DmsD3 | Desor_3110 | YP_004971142 |
| Firmicutes | Clostridia | *Desulfosporosinus orientis DSM 765* | DmsD1 | Desor_3142 | YP_004971169 |
| Firmicutes | Clostridia | *Desulfosporosinus orientis DSM 765* | DmsD2 | Desor_2560 | AET68114 |
| Firmicutes | Clostridia | *Desulfosporosinus orientis DSM 765* | YcdY | Desor_2604 | AET68154 |
| Firmicutes | Clostridia | *Symbiobacterium thermophilum IAM 14863* | DmsD1 | STH2333 | YP_076162 |
| Firmicutes | Clostridia | *Symbiobacterium thermophilum IAM 14863* | DmsD2 | STH710 | YP_074539 |
| Firmicutes | Clostridia | *Thermodesulfobium narugense DSM 14796* | NarJ | Thena_0096 | AEE13747 |
| Firmicutes | Negativicutes | *Selenomonas sputigena ATCC 35185* | NarJ | Selsp_0712 | AEB99679 |
| Firmicutes | Negativicutes | *Veillonella parvula DSM 2008* | NarJ | Vpar_0224 | ACZ23911 |
| ***Gram-negatives*** | | | | | |
| Proteobacteria | Alpha-proteobacteria | *Acidiphilium cryptum JF-5* | NarJ | Acry_1583 | ABQ30789 |
| Proteobacteria | Alpha-proteobacteria | *Azorhizobium caulinodans ORS 571* | NarJ | AZC_1427 | YP_001524343 |
| Proteobacteria | Alpha-proteobacteria | *Azospirillum lipoferum 4B* | DmsD | AZOLI_0632 | CBS85999 |
| Proteobacteria | Alpha-proteobacteria | *Azospirillum lipoferum 4B* | TorD | AZOLI_2296 | CBS87514 |
| Proteobacteria | Alpha-proteobacteria | *Azospirillum lipoferum 4B* | DmsDp | AZOLI_p40295 | CBS90684 |
| Proteobacteria | Alpha-proteobacteria | *Bradyrhizobium japonicum USDA 6* | DmsD1 | BJ6T_15590 | YP_005606429 |
| Proteobacteria | Alpha-proteobacteria | *Bradyrhizobium japonicum USDA 6* | DmsD2 | BJ6T_06980 | YP_005605580 |
|  |  |  |  |  |  |
| **Phylum** | **Class** | **Genus and species** | **NarJ sub-family** | **Gene locus tag** | **Protein accession number** |
| Proteobacteria | Alpha-proteobacteria | *Chelativorans sp. BNC1* | DmsD | Meso_2447 | ABG63831 |
| Proteobacteria | Alpha-proteobacteria | *Hyphomicrobium denitrificans ATCC 51888* | NarJ | Hden_0928 | ADJ22742 |
| Proteobacteria | Alpha-proteobacteria | *Magnetospirillum magneticum AMB-1* | TorD | amb2669 | BAE51473 |
| Proteobacteria | Alpha-proteobacteria | *Methylobacterium extorquens DM4* | TorD | METDI0452 | CAX22111 |
| Proteobacteria | Alpha-proteobacteria | *Micavibrio aeruginosavorus EPB* | NarJ | A11S_831 | AGH97654 |
| Proteobacteria | Alpha-proteobacteria | *Nitrobacter hamburgensis X14* | NarJ | Nham_3446 | ABE64175 |
| Proteobacteria | Alpha-proteobacteria | *Nitrobacter hamburgensis X14* | TorD | Nham_3124 | ABE63860 |
| Proteobacteria | Alpha-proteobacteria | *Ochrobactrum anthropi ATCC 49188* | TorD | Oant_0220 | ABS12951 |
| Proteobacteria | Alpha-proteobacteria | *Ochrobactrum anthropi ATCC 49188* | NarJ | Oant_2893 | ABS15601 |
| Proteobacteria | Alpha-proteobacteria | *Phaeobacter gallaeciensis 2. 10* | TorD | PGA1_c27670 | AFO92434 |
| Proteobacteria | Alpha-proteobacteria | *Phaeobacter gallaeciensis 2. 10* | TorD2 | PGA2_c25680 | AFO88550 |
| Proteobacteria | Alpha-proteobacteria | *Pseudovibrio sp. FO-BEG1* | TorD1 | PSE_3936 | AEV38440 |
| Proteobacteria | Alpha-proteobacteria | *Pseudovibrio sp. FO-BEG1* | TorD2 | PSE_4526 | AEV39028 |
| Proteobacteria | Alpha-proteobacteria | *Pseudovibrio sp. FO-BEG1* | NarJ | PSE_0759 | AEV35271 |
| Proteobacteria | Alpha-proteobacteria | *Rhodobacter capsulatus SB 1003* | TorD | RCAP_rcc02846 | YP_003578982 |
| Proteobacteria | Alpha-proteobacteria | *Roseobacter denitrificans OCh 114* | TorD | RD1_2899 | ABG32423 |
| Proteobacteria | Alpha-proteobacteria | *Roseobacter denitrificans OCh 114* | NarJ | RD1_2444 | YP_682696 |
| Proteobacteria | Alpha-proteobacteria | *Sinorhizobium medicae WSM419* | TorD | Smed_5493 | ABR64247 |
| Proteobacteria | Beta-proteobacteria | *Achromobacter xylosoxidans NH44784-1996* | NarJ | NH44784_049251 | CCH08868 |
| Proteobacteria | Beta-proteobacteria | *Achromobacter xylosoxidans NH44784-1996* | TorD | NH44784_040581 | CCH08002 |
| Proteobacteria | Beta-proteobacteria | *Alicycliphilus denitrificans K601* | NarJ | Alide2_0467 | AEB82886 |
| Proteobacteria | Beta-proteobacteria | *Alicycliphilus denitrificans K601* | TorD | Alide2_1050 | AEB83457 |
| Proteobacteria | Beta-proteobacteria | *Burkholderia glumae BGR1* | NarJ | bglu_2g17410 | ACR32082 |
| Proteobacteria | Beta-proteobacteria | *Burkholderia glumae BGR1* | TorD | bglu_2g01860 | ACR30662 |
| Proteobacteria | Beta-proteobacteria | *Burkholderia glumae BGR1* | DmsD | bglu_1g12340 | ACR28393 |
|  |  |  |  |  |  |
| **Phylum** | **Class** | **Genus and species** | **NarJ sub-family** | **Gene locus tag** | **Protein accession number** |
| Proteobacteria | Beta-proteobacteria | *Chromobacterium violaceum ATCC 12472* | NarJ | CV_2541 | AAQ60212 |
| Proteobacteria | Beta-proteobacteria | *Delftia acidovorans SPH-1* | NarJ | Daci_1111 | ABX33756 |
| Proteobacteria | Beta-proteobacteria | *Laribacter hongkongensis HLHK9* | DmsD | LHK_02499 | ACO75481 |
| Proteobacteria | Beta-proteobacteria | *Ralstonia eutropha JMP134* | NarJ | Reut_B5004 | AAZ64352 |
| Proteobacteria | Beta-proteobacteria | *Ralstonia eutropha JMP134* | TorD | Reut_A0680 | AAZ60062 |
| Proteobacteria | Beta-proteobacteria | *Rubrivivax gelatinosus IL144* | DmsD | RGE_20290 | BAL95370 |
| Proteobacteria | Beta-proteobacteria | *Rubrivivax gelatinosus IL144* | TorD | RGE_07280 | BAL94071 |
| Proteobacteria | Beta-proteobacteria | *Thauera sp. MZ1T* | NarJ | Tmz1t_2633 | ACR01235 |
| Proteobacteria | Beta-proteobacteria | *Thauera sp. MZ1T* | TorD1 | Tmz1t_3711 | ACR02304 |
| Proteobacteria | Beta-proteobacteria | *Thauera sp. MZ1T* | TorD2 | Tmz1t_2732 | ACR01333 |
| Proteobacteria | Beta-proteobacteria | *Thauera sp. MZ1T* | Nar | Tmz1t_0388 | ACK53170 |
| Proteobacteria | Beta-proteobacteria | *Thauera sp. MZ1T* | DmsD | Tmz1t_0360 | ACK53144 |
| Proteobacteria | Gamma-proteobacteria | *Acidithiobacillus caldus SM-1* | NarJ | Atc_1936A | AEK59531 |
| Proteobacteria | Gamma-proteobacteria | *Actinobacillus succinogenes 130Z* | DmsD | Asuc_1520 | YP_001344813 |
| Proteobacteria | Gamma-proteobacteria | *Actinobacillus succinogenes 130Z* | TorD1 | Asuc_0999 | YP_001344302 |
| Proteobacteria | Gamma-proteobacteria | *Actinobacillus succinogenes 130Z* | TorD2 | Asuc_0899 | YP_001344204 |
| Proteobacteria | Gamma-proteobacteria | *Aeromonas hydrophila ML09-119* | TorD1 | AHML_17840 | AGM45334 |
| Proteobacteria | Gamma-proteobacteria | *Aeromonas hydrophila ML09-119* | TorD2 | AHML_16525 | AGM45073 |
| Proteobacteria | Gamma-proteobacteria | *Aeromonas hydrophila ML09-119* | TorD3 | AHML_15855 | AGM44939 |
| Proteobacteria | Gamma-proteobacteria | *Aggregatibacter actinomycetemcomitans D11S-1* | TorD1 | D11S_1669 | ACX83032 |
| Proteobacteria | Gamma-proteobacteria | *Aggregatibacter actinomycetemcomitans D11S-1* | TorD2 | D7S_02426 | AFI88117 |
| Proteobacteria | Gamma-proteobacteria | *Aggregatibacter actinomycetemcomitans D11S-1* | DmsD1 | D11S_0496 | ACX81906 |
| Proteobacteria | Gamma-proteobacteria | *Aggregatibacter actinomycetemcomitans D11S-1* | DmsD2 | D7S_01379 | AFI87144 |
| Proteobacteria | Gamma-proteobacteria | *Alcanivorax dieselolei B5* | NarJ | B5T_03061 | AFT71329 |
| Proteobacteria | Gamma-proteobacteria | *Alteromonas macleodii str. 'Deep ecotype'* | NarJ1 | MADE_1019265 | AEA99975 |
|  |  |  |  |  |  |
| **Phylum** | **Class** | **Genus and species** | **NarJ sub-family** | **Gene locus tag** | **Protein accession number** |
| Proteobacteria | Gamma-proteobacteria | *Bibersteinia trehalosi USDA-ARS-USMARC-192* | DmsD | WQG_10810 | AGH38358 |
| Proteobacteria | Gamma-proteobacteria | *Bibersteinia trehalosi USDA-ARS-USMARC-192* | TorD | WQG_16210 | AGH38898 |
| Proteobacteria | Gamma-proteobacteria | *Chromohalobacter salexigens DSM 3043* | TorD | Csal_1913 | ABE59265 |
| Proteobacteria | Gamma-proteobacteria | *Chromohalobacter salexigens DSM 3043* | NarJ | Csal_1332 | ABE58687 |
| Proteobacteria | Gamma-proteobacteria | *Citrobacter koseri ATCC BAA-895* | NarJ1 | CKO_01303 | ABV12443 |
| Proteobacteria | Gamma-proteobacteria | *Citrobacter koseri ATCC BAA-895* | NarJ2 | CKO_01501 | ABV12634 |
| Proteobacteria | Gamma-proteobacteria | *Citrobacter koseri ATCC BAA-895* | DmsD | CKO_01585 | ABV12717 |
| Proteobacteria | Gamma-proteobacteria | *Citrobacter koseri ATCC BAA-895* | TorD | CKO_02034 | ABV13160 |
| Proteobacteria | Gamma-proteobacteria | *Colwellia psychrerythraea 34H* | TorD | CPS_1834 | AAZ24720 |
| Proteobacteria | Gamma-proteobacteria | *Cronobacter sakazakii ATCC BAA-894* | NarJ | ESA_01522 | ABU76780 |
| Proteobacteria | Gamma-proteobacteria | *Cronobacter sakazakii ATCC BAA-894* | torD | ESA_02302 | ABU77551 |
| Proteobacteria | Gamma-proteobacteria | *Cronobacter sakazakii ATCC BAA-894* | DmsD | ESA_01743 | ABU76997 |
| Proteobacteria | Gamma-proteobacteria | *Dickeya dadantii Ech586* | DmsD | Dd586_0251 | ACZ75148 |
| Proteobacteria | Gamma-proteobacteria | *Dickeya dadantii Ech586* | TorD | Dd586_2294 | ACZ77145 |
| Proteobacteria | Gamma-proteobacteria | *Dickeya dadantii Ech586* | NarJ | Dd586_1820 | ACZ76683 |
| Proteobacteria | Gamma-proteobacteria | *Edwardsiella tarda EIB202* | NarJ | ETAE_0249 | ACY83096 |
| Proteobacteria | Gamma-proteobacteria | *Edwardsiella tarda EIB202* | DmsD | ETAE_2191 | ACY85026 |
| Proteobacteria | Gamma-proteobacteria | *Edwardsiella tarda EIB202* | TorD | ETAE_1420 | ACY84261 |
| Proteobacteria | Gamma-proteobacteria | *Edwardsiella tarda EIB202* | TorD1 | ETAE_0297 | ACY83144 |
| Proteobacteria | Gamma-proteobacteria | *Escherichia coli str. K-12 substr. MG1655* | NarJ | b1226 | BAL38294 |
| Proteobacteria | Gamma-proteobacteria | *Escherichia coli str. K-12 substr. MG1655* | NarW | b1466 | AAC74548 |
| Proteobacteria | Gamma-proteobacteria | *Escherichia coli str. K-12 substr. MG1655* | TorD | b0998 | AAC74083 |
| Proteobacteria | Gamma-proteobacteria | *Escherichia coli str. K-12 substr. MG1655* | DmsD | b1591 | AAC74663 |
| Proteobacteria | Gamma-proteobacteria | *Escherichia coli str. K-12 substr. MG1655* | YcdY | b1035 | AAC74119 |
| Proteobacteria | Gamma-proteobacteria | *Gallibacterium anatis UMN179* | DmsD1 | UMN179_00120 | AEC16157 |
|  |  |  |  |  |  |
| **Phylum** | **Class** | **Genus and species** | **NarJ sub-family** | **Gene locus tag** | **Protein accession number** |
| Proteobacteria | Gamma-proteobacteria | *Gallibacterium anatis UMN179* | DmsD2 | UMN179_01190 | AEC17213 |
| Proteobacteria | Gamma-proteobacteria | *Haemophilus parainfluenzae T3T1* | TorD | PARA_19280 | CBW16028 |
| Proteobacteria | Gamma-proteobacteria | *Halomonas elongata DSM 2581* | NarJ | HELO_2852 | CBV42736 |
| Proteobacteria | Gamma-proteobacteria | *Klebsiella pneumoniae JM45* | NarJ1 | N559_2069 | AGT23788 |
| Proteobacteria | Gamma-proteobacteria | *Klebsiella pneumoniae JM45* | NarJ2 | N559_2408 | AGT24104 |
| Proteobacteria | Gamma-proteobacteria | *Klebsiella pneumoniae JM45* | TorD | N559_3231 | AGT24891 |
| Proteobacteria | Gamma-proteobacteria | *Klebsiella pneumoniae JM45* | DmsD | N559_2748 | AGT24421 |
| Proteobacteria | Gamma-proteobacteria | *Mannheimia succiniciproducens MBEL55E* | TorD | MS0837 | AAU37444 |
| Proteobacteria | Gamma-proteobacteria | *Mannheimia succiniciproducens MBEL55E* | DmsD | MS2335 | AAU38942 |
| Proteobacteria | Gamma-proteobacteria | *Morganella morganii subsp. morganii KT* | NarJ | MU9_69 | AGG29115 |
| Proteobacteria | Gamma-proteobacteria | *Morganella morganii subsp. morganii KT* | DmsD1 | MU9_1149 | AGG30195 |
| Proteobacteria | Gamma-proteobacteria | *Morganella morganii subsp. morganii KT* | DmsD2 | MU9_398 | AGG30190 |
| Proteobacteria | Gamma-proteobacteria | *Morganella morganii subsp. morganii KT* | DmsD3 | MU9_1144 | AGG29444 |
| Proteobacteria | Gamma-proteobacteria | *Nitrosococcus halophilus Nc 4* | TorD | Nhal_1621 | ADE14755 |
| Proteobacteria | Gamma-proteobacteria | *Pantoea ananatis LMG 5342* | YcdY | PANA5342_2664 | CCF10057 |
| Proteobacteria | Gamma-proteobacteria | *Pectobacterium carotovorum subsp. carotovorum PC1* | NarJ | PC1_2257 | ACT13295 |
| Proteobacteria | Gamma-proteobacteria | *Pectobacterium carotovorum subsp. carotovorum PC1* | TorD | PC1_1799 | ACT12840 |
| Proteobacteria | Gamma-proteobacteria | *Photobacterium profundum SS9* | NarJ1 | PBPRA1864 | CAG20268 |
| Proteobacteria | Gamma-proteobacteria | *Photobacterium profundum SS9* | NarJ | PBPRB0331 | CAG22204 |
| Proteobacteria | Gamma-proteobacteria | *Photobacterium profundum SS9* | TorD | PBPRA1494 | CAG19905 |
| Proteobacteria | Gamma-proteobacteria | *Proteus mirabilis BB2000* | DmsD | BB2000_1242 | AGS59735 |
| Proteobacteria | Gamma-proteobacteria | *Proteus mirabilis BB2000* | TorD | BB2000_1135 | AGS59628 |
| Proteobacteria | Gamma-proteobacteria | *Proteus mirabilis BB2000* | NarJ | BB2000_0015 | AGS58533 |
| Proteobacteria | Gamma-proteobacteria | *Providencia stuartii MRSN 2154* | NarJ | S70_07640 | AFH93397 |
| Proteobacteria | Gamma-proteobacteria | *Providencia stuartii MRSN 2154* | DmsD | S70_16235 | AFH95817 |
|  |  |  |  |  |  |
| **Phylum** | **Class** | **Genus and species** | **NarJ sub-family** | **Gene locus tag** | **Protein accession number** |
| Proteobacteria | Gamma-proteobacteria | *Providencia stuartii MRSN 2154* | TorD | S70_20160 | AFH95817 |
| Proteobacteria | Gamma-proteobacteria | *Providencia stuartii MRSN 2154* | DmsD2 | S70_12815 | AFH94406 |
| Proteobacteria | Gamma-proteobacteria | *Pseudomonas aeruginosa PAO1* | NarJ | PA3873 | AE004804 |
| Proteobacteria | Gamma-proteobacteria | *Psychrobacter sp. PRwf-1* | NarJ | PsycPRwf_0668 | ABQ93622 |
| Proteobacteria | Gamma-proteobacteria | *Psychrobacter sp. PRwf-1* | TorD | PsycPRwf_0215 | ABQ93174 |
| Proteobacteria | Gamma-proteobacteria | *Rahnella sp. Y9602* | NarJ | Rahaq_2440 | ADW74047 |
| Proteobacteria | Gamma-proteobacteria | *Rahnella sp. Y9602* | TorD1 | Rahaq_1693 | ADW73311 |
| Proteobacteria | Gamma-proteobacteria | *Rahnella sp. Y9602* | TorD2 | Rahaq_1459 | ADW73081 |
| Proteobacteria | Gamma-proteobacteria | *Salmonella enterica subsp. enterica serovar Typhimurium str. LT2* | NarJ | STM1762 | AAL20677 |
| Proteobacteria | Gamma-proteobacteria | *Salmonella enterica subsp. enterica serovar Typhimurium str. LT2* | NarW | STM1579 | AAL20497 |
| Proteobacteria | Gamma-proteobacteria | *Salmonella enterica subsp. enterica serovar Typhimurium str. LT2* | TorD | STM3821 | AAL22680 |
| Proteobacteria | Gamma-proteobacteria | *Salmonella enterica subsp. enterica serovar Typhimurium str. LT2* | DmsD | STM1495 | AAL20414 |
| Proteobacteria | Gamma-proteobacteria | *Salmonella enterica subsp. enterica serovar Typhimurium str. LT2* | DmsD2/TorD | STM0610 | AAL19561 |
| Proteobacteria | Gamma-proteobacteria | *Salmonella enterica subsp. enterica serovar Typhimurium str. LT2* | DmsD3 | STM4308 | AAL23132 |
| Proteobacteria | Gamma-proteobacteria | *Serratia proteamaculans 568* | NarJ | Spro_2881 | ABV41982 |
| Proteobacteria | Gamma-proteobacteria | *Serratia proteamaculans 568* | TorD1 | Spro_4217 | ABV43311 |
| Proteobacteria | Gamma-proteobacteria | *Serratia proteamaculans 568* | DmsD | Spro_1690 | ABV40794 |
| Proteobacteria | Gamma-proteobacteria | *Serratia proteamaculans 568* | TorD2 | Spro_1935 | ABV41038 |
| Proteobacteria | Gamma-proteobacteria | *Shewanella sediminis HAW-EB3* | NarJ | Ssed_1956 | ABV36565 |
| Proteobacteria | Gamma-proteobacteria | *Shewanella sediminis HAW-EB3* | DmsD1 | Ssed_0231 | ABV34844 |
| Proteobacteria | Gamma-proteobacteria | *Shewanella sediminis HAW-EB3* | DmsD2 | Ssed_0356 | ABV34969 |
| Proteobacteria | Gamma-proteobacteria | *Shewanella sediminis HAW-EB3* | TorD1 | Ssed_4423 | ABV39025 |
| Proteobacteria | Gamma-proteobacteria | *Shewanella sediminis HAW-EB3* | TorD2 | Ssed_4213 | ABV38817 |
| Proteobacteria | Gamma-proteobacteria | *Shewanella sediminis HAW-EB3* | TorD3 | Ssed_0348 | ABV34961 |
| Proteobacteria | Gamma-proteobacteria | *Shewanella sediminis HAW-EB3* | DmsD3 | Ssed_2921 | ABV37528 |
|  |  |  |  |  |  |
| **Phylum** | **Class** | **Genus and species** | **NarJ sub-family** | **Gene locus tag** | **Protein accession number** |
| Proteobacteria | Gamma-proteobacteria | *Shewanella sediminis HAW-EB3* | DmsD4 | Ssed_1406 | ABV36017 |
| Proteobacteria | Gamma-proteobacteria | *Shewanella sediminis HAW-EB3* | TorD4 | Ssed_3361 | ABV37965 |
| Proteobacteria | Gamma-proteobacteria | *Shewanella sediminis HAW-EB3* | TorD5 | Ssed_1309 | ABV35920 |
| Proteobacteria | Gamma-proteobacteria | *Stenotrophomonas maltophilia K279a* | NarJ | Smlt2772 | CAQ46239 |
| Proteobacteria | Gamma-proteobacteria | *Vibrio alginolyticus NBRC 15630 = ATCC 17749* | TorD1 | N646_0095 | AGV15928 |
| Proteobacteria | Gamma-proteobacteria | *Vibrio alginolyticus NBRC 15630 = ATCC 17749* | TorD2 | N646_0565 | AGV16398 |
| Proteobacteria | Gamma-proteobacteria | *Xenorhabdus bovienii SS-2004* | TorD | XBJ1_2214 | CBJ81340 |
| Proteobacteria | Gamma-proteobacteria | *Yersinia enterocolitica subsp. palearctica 105.5R* | TorD1 | YE105_C0685 | ADZ41181 |
| Proteobacteria | Gamma-proteobacteria | *Yersinia enterocolitica subsp. palearctica 105.5R(r* | TorD2 | YE105_C1839 | ADZ42335 |
| Proteobacteria | Delta-proteobacteria | *Anaeromyxobacter dehalogenans 2CP-C* | NarJ | Adeh_2172 | ABC81942 |
| Proteobacteria | Delta-proteobacteria | *Desulfobacterium autotrophicum HRM2* | NarJ | HRM2_00640 | ACN13187 |
| Proteobacteria | Delta-proteobacteria | *Desulfobulbus propionicus DSM 2032* | TorD | Despr_2398 | ADW18539 |
| Proteobacteria | Delta-proteobacteria | *Desulfomonile tiedjei DSM 6799* | NarJ | Desti_0966 | AFM23684 |
| Proteobacteria | Delta-proteobacteria | *Desulfomonile tiedjei DSM 6799* | TorD | Desti_1384 | AFM24096 |
| Proteobacteria | Delta-proteobacteria | *Desulfovibrio salexigens DSM 2638* | TorD | Desal_0377 | ACS78444 |
| Proteobacteria | Delta-proteobacteria | *Desulfurivibrio alkaliphilus AHT2* | TorD1 | DaAHT2_0206 | ADH84917 |
| Proteobacteria | Delta-proteobacteria | *Desulfurivibrio alkaliphilus AHT2* | TorD2 | DaAHT2_0421 | ADH85127 |
| Proteobacteria | Delta-proteobacteria | *Geobacter lovleyi SZ* | NarJ2 | Glov_2654 | ACD93936 |
| Proteobacteria | Delta-proteobacteria | *Geobacter lovleyi SZ* | NarJ1 | Glov_0205 | ACD96367 |
| Proteobacteria | Delta-proteobacteria | *Geobacter lovleyi SZ* | TorD | Glov_1147 | ACD94869 |
| Proteobacteria | Epsilon-proteobacteria | *Arcobacter nitrofigilis DSM 7299* | DmsD | Arnit_0975 | ADG92639 |
| Proteobacteria | Epsilon-proteobacteria | *Arcobacter nitrofigilis DSM 7299* | TorD | Arnit_0968 | ADG92632 |
| Proteobacteria | Epsilon-proteobacteria | *Campylobacter curvus 525.92* | TorD1 | CCV52592_2104 | EAU00845 |
| Proteobacteria | Epsilon-proteobacteria | *Campylobacter curvus 525.92* | TorD2 | CCV52592_0530 | EAU00726 |
| Proteobacteria | Epsilon-proteobacteria | *Sulfuricurvum kujiense DSM 16994* | NarJ1 | Sulku_2487 | ADR35146 |
|  |  |  |  |  |  |
| **Phylum** | **Class** | **Genus and species** | **NarJ sub-family** | **Gene locus tag** | **Protein accession number** |
| Proteobacteria | Epsilon-proteobacteria | *Sulfuricurvum kujiense DSM 16994* | NarJ2 | Sulku_0643 | ADR33309 |
| Proteobacteria | Epsilon-proteobacteria | *Sulfurospirillum barnesii SES-3* | DmsD | Sulba_0897 | AFL68198 |
| Proteobacteria | Epsilon-proteobacteria | *Sulfurospirillum barnesii SES-3* | DmsD2 | Sulba_0857 | AFL68158 |
| Proteobacteria | Epsilon-proteobacteria | *Nitratiruptor sp. SB155-2* | NarJ1 | NIS_0577 | BAF69691 |
| Proteobacteria | Epsilon-proteobacteria | *Nitratiruptor sp. SB155-2* | NarJ2 | NIS_1815 | BAF70920 |
| Proteobacteria | Epsilon-proteobacteria | *Wolinella succinogenes DSM 1740* | Nar | WS1144 | CAE10231 |
| Proteobacteria | Epsilon-proteobacteria | *Wolinella succinogenes DSM 1740* | DmsD | WS1433 | CAE10494 |
| Proteobacteria | Epsilon-proteobacteria | *Wolinella succinogenes DSM 1740* | TorD | WS0731 | CAE09851 |
| Chlorobi | Chlorobia | *Pelodictyon phaeoclathratiforme BU-1* | TorD | Ppha_1338 | ACF43602 |
| Chlorobi | Chlorobia | *Chloroherpeton thalassium ATCC 35110* | TorD | Ctha_0876 | ACF13344 |
| Chlorobi | Chlorobia | *Chloroherpeton thalassium ATCC 35110* | REMP | Ctha_0120 | ACF12591 |
| Chlorobi | Chlorobia | *Chlorobaculum parvum NCIB 8327* | TorD | Cpar_1391 | ACF11792 |
| Chlorobi | Chlorobia | *Chlorobium phaeobacteroides BS1* | TorD | Cphamn1_1866 | ACE04783 |
| Chrysiogenetes | Chrysiogenetes | *Desulfurispirillum indicum S5* | NarJ | Selin_2441 | ADU67156 |
| Chrysiogenetes | Chrysiogenetes | *Desulfurispirillum indicum S5* | TorD | Selin_0380 | ADU65136 |
| Chrysiogenetes | Chrysiogenetes | *Desulfurispirillum indicum S5* | DmsD | Selin_2610 | ADU67321 |
| Chrysiogenetes | Chrysiogenetes | *Desulfurispirillum indicum S5* | REMP | Selin_2178 | ADU66898 |
| Deferribacteres | Deferribacteres | *Denitrovibrio acetiphilus DSM 12809* | REMP1/ TorD | Dacet_2621 | ADD69379 |
| Deferribacteres | Deferribacteres | *Denitrovibrio acetiphilus DSM 12809* | NarJ | Dacet_2068 | ADD68831 |
| Deferribacteres | Deferribacteres | *Denitrovibrio acetiphilus DSM 12809* | TorD1 | Dacet_2171 | ADD68933 |
| Deferribacteres | Deferribacteres | *Denitrovibrio acetiphilus DSM 12809* | TorD2 | Dacet_2123 | ADD68885 |
| Deferribacteres | Deferribacteres | *Denitrovibrio acetiphilus DSM 12809* | REMP2 | Dacet_1680 | ADD68444 |
| Deferribacteres | Deferribacteres | *Denitrovibrio acetiphilus DSM 12809* | TorD3 | Dacet_0681 | ADD67468 |
| Deferribacteres | Deferribacteres | *Denitrovibrio acetiphilus DSM 12809* | REMP3 | Dacet_0207 | ADD67011 |
| Deferribacteres | Deferribacteres | *Denitrovibrio acetiphilus DSM 12809* | DmsD | Dacet_2576 | ADD69335 |
|  |  |  |  |  |  |
| **Phylum** | **Class** | **Genus and species** | **NarJ sub-family** | **Gene locus tag** | **Protein accession number** |
| Deferribacteres | Deferribacteres | *Denitrovibrio acetiphilus DSM 12809* | REMP5 | Dacet_2101 | ADD68864 |
| Deferribacteres | Deferribacteres | *Denitrovibrio acetiphilus DSM 12809* | REMP6 | Dacet_0685 | ADD67471 |
| Deferribacteres | Deferribacteres | *Calditerrivibrio nitroreducens DSM 19672* | TorD | Calni_0808 | ADR18719 |
| Deferribacteres | Deferribacteres | *Deferribacter desulfuricans SSM1* | NarJ | DEFDS_2087 | BAI81535 |
| Deferribacteres | Deferribacteres | *Deferribacter desulfuricans SSM1* | TorD | DEFDS_1625 | BAI81083 |
| Deferribacteres | Deferribacteres | *Deferribacter desulfuricans SSM1* | TorD2 | DEFDS_1333 | BAI80796 |
| Deferribacteres | Deferribacteres | *Flexistipes sinusarabici DSM 4947* | TorD1 | Flexsi_1175 | AEI14830 |
| Deferribacteres | Deferribacteres | *Flexistipes sinusarabici DSM 4947* | TorD2 | Flexsi_2262 | AEI15881 |
| Deferribacteres | Deferribacteres | *Flexistipes sinusarabici DSM 4947* | REMP | Flexsi_1072 | AEI14729 |
| **Archaea** | | | | | |
| Archaea | Euryarchaea | *Archaeoglobus fulgidus DSM 4304* | YcdY? | AF0160 | AAB91075 |
| Archaea | Euryarchaea | *Archaeoglobus fulgidus DSM 4304* | REMP/NarJ | AF0173 | AAB91059 |
| Archaea | Euryarchaea | *Archaeoglobus veneficus SNP6* | REMP1 | Arcve_1246 | AEA47253 |
| Archaea | Euryarchaea | *Archaeoglobus veneficus SNP6* | REMP2 | Arcve_0719 | AEA46738 |
| Archaea | Euryarchaea | *Ferroglobus placidus DSM 10642* | NarJ | Ferp_0313 | ADC64493 |
| Archaea | Euryarchaea | *Halorubrum lacusprofundi ATCC 49239* | DmsD | Hlac_1338 | ACM56930 |
| Archaea | Euryarchaea | *Haloterrigena turkmenica DSM 5511* | REMP1 | Htur_1651 | ADB60536 |
| Archaea | Euryarchaea | *Halomicrobium mukohataei DSM 12286* | DmsD | Hmuk_2467 | ACV48575 |
| Archaea | Euryarchaea | *Natronobacterium gregoryi SP2* | REMP1 | Natgr_3307 | AFZ74432 |
| Archaea | Euryarchaea | *Natronobacterium gregoryi SP2* | REMP2 | Natgr_2279 | AFZ73455 |
| Archaea | Euryarchaea | *Natronobacterium gregoryi SP2* | REMP3 | Natgr_0554 | AFZ71805 |
| Archaea | Euryarchaea | *Natronobacterium gregoryi SP2* | REMP4 | Natgr_0410 | AFZ71665 |
| Archaea | Euryarchaea | *Natronomonas pharaonis DSM 2160* | REMP1 | NP2586A | CAI49384 |
| Archaea | Euryarchaea | *Natronomonas pharaonis DSM 2160* | REMP2 | NP4956A | CAI50569 |
| Archaea | Euryarchaea | *Halomicrobium mukohataei DSM 12286* | TorD1 | Hmuk_1563 | ACV47678 |
| Archaea | Euryarchaea | *Halomicrobium mukohataei DSM 12286* | TorD2 | Hmuk_3261 | ACV49361 |
| Archaea | Euryarchaea | *Haloterrigena turkmenica DSM 5511* | REMP2 | Htur_2354 | ADB61232 |
| **Phylum** | **Class** | **Genus and species** | **NarJ sub-family** | **Gene locus tag** | **Protein accession number** |
| Archaea | Euryarchaea | *Ferroglobus placidus DSM 10642* | REMP1 | Ferp_1008 | ADC65170 |
| Archaea | Euryarchaea | *Ferroglobus placidus DSM 10642* | REMP2 | Ferp_0121 | ADC64309 |
| Archaea | Euryarchaea | *Ferroglobus placidus DSM 10642* | REMP3 | Ferp_1254 | ADC65408 |
| Archaea | Chrenarchaea | *Acidianus hospitalis W1* | DmsD | Ahos_0233 | AEE93125 |
| Archaea | Chrenarchaea | *Aeropyrum camini SY1 = JCM 12091* | NarJ | ACAM_0822 | BAN90291 |
| Archaea | Chrenarchaea | *Pyrobaculum sp. 1860* | NarJ | P186_0776 | AET32222 |
| Archaea | Chrenarchaea | *Sulfolobus islandicus M.16.27* | NarJ | M1627_0834 | ACP54777 |
| Archaea | Chrenarchaea | *Sulfolobus islandicus M.16.27* | TorD | M1627_0543 | ACP54529 |
| Archaea | Chrenarchaea | *Vulcanisaeta distributa DSM 14429* | NarJ | Vdis_1439 | ADN50825 |
| Archaea | Chrenarchaea | *Vulcanisaeta distributa DSM 14429* | TorD | Vdis_2164 | ADN51533 |
| Archaea | Chrenarchaea | *Thermoproteus tenax Kra 1* | TorD | TTX_0048 | CCC80726 |
| Archaea | Chrenarchaea | *Pyrobaculum sp. 1860* | TorD | P186_2712 | AET34096 |
| Archaea | Chrenarchaea | *Sulfolobus islandicus M.16.27* | TorD2 | M1627_2573 | ACP56418 |
|  |  |  |  |  |  |
