# Supplementary figures and images for: NarJ subfamily system specific chaperone diversity and evolution is directed by respiratory enzyme associations
Source: BMC Evol Biol. 2015 Jun 12;15:110. doi: 10.1186/s12862-015-0412-3 (PMC4464133; doi:10.1186/s12862-015-0412-3)

## Figure S1

## NarJ subfamily protein multiple sequence alignment

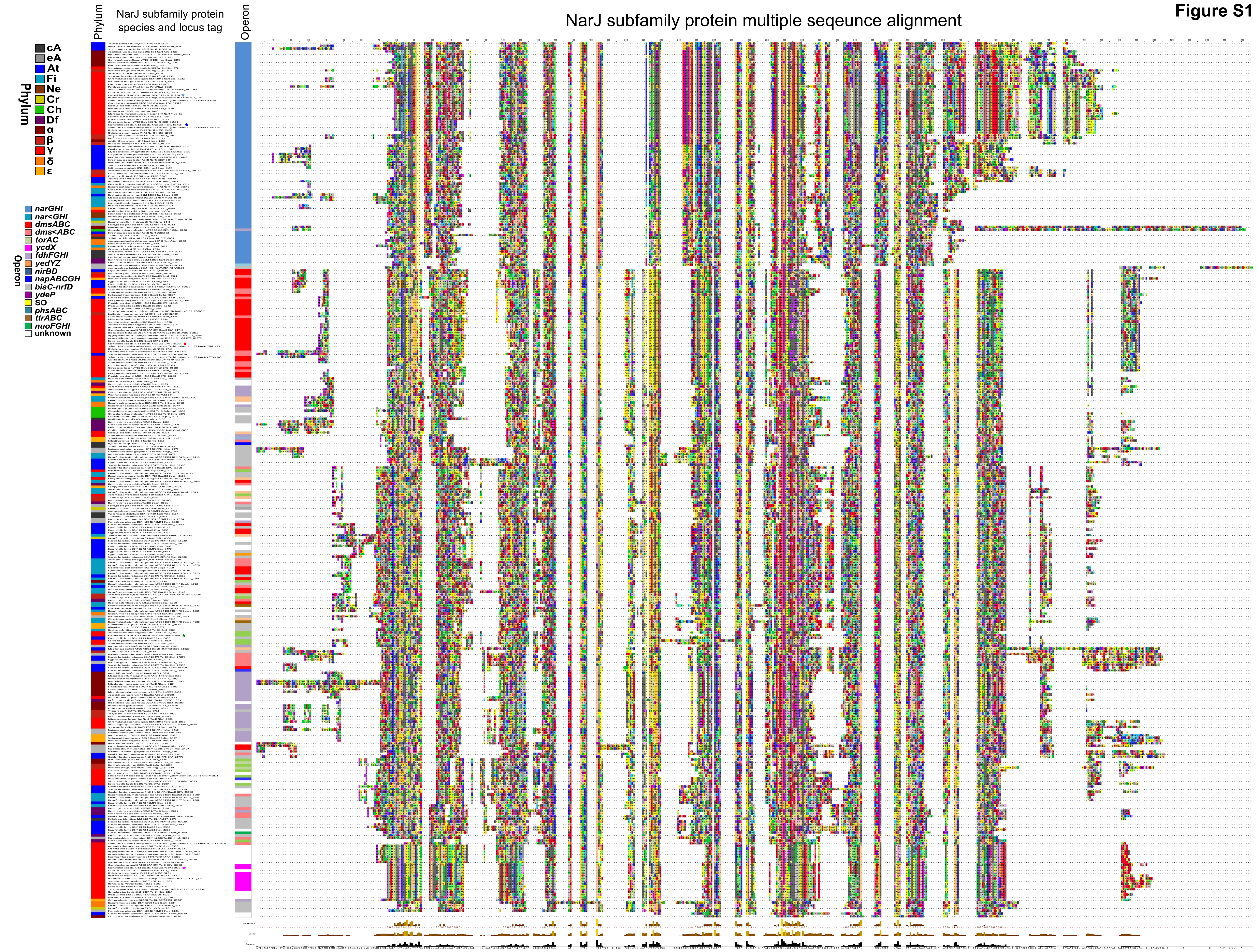

Supplement: Additional file 2: Figure S1. — An unedited multiple sequence alignment (MSA) of 324 NarJ subfamily proteins examined in this study. The MSA shown represents a consensus between Cobalt [38] and Praline [39] MSA programs. All protein sequences shown in the alignment are unedited. Positions with >80 % gaps were removed from this alignment to perform phylogenetic analysis and Sd/Nd analysis to avoid excessive gap penalties and prevent analysis errors caused by gaps. Each sequence is labelled according to its species, NarJ subfamily annotation (according to tBLASTn e-values < 1.0 × 10^−4^) and its respective Genbank/ NCBI Gene locus tag. [file 12862_2015_412_MOESM2_ESM.pdf]

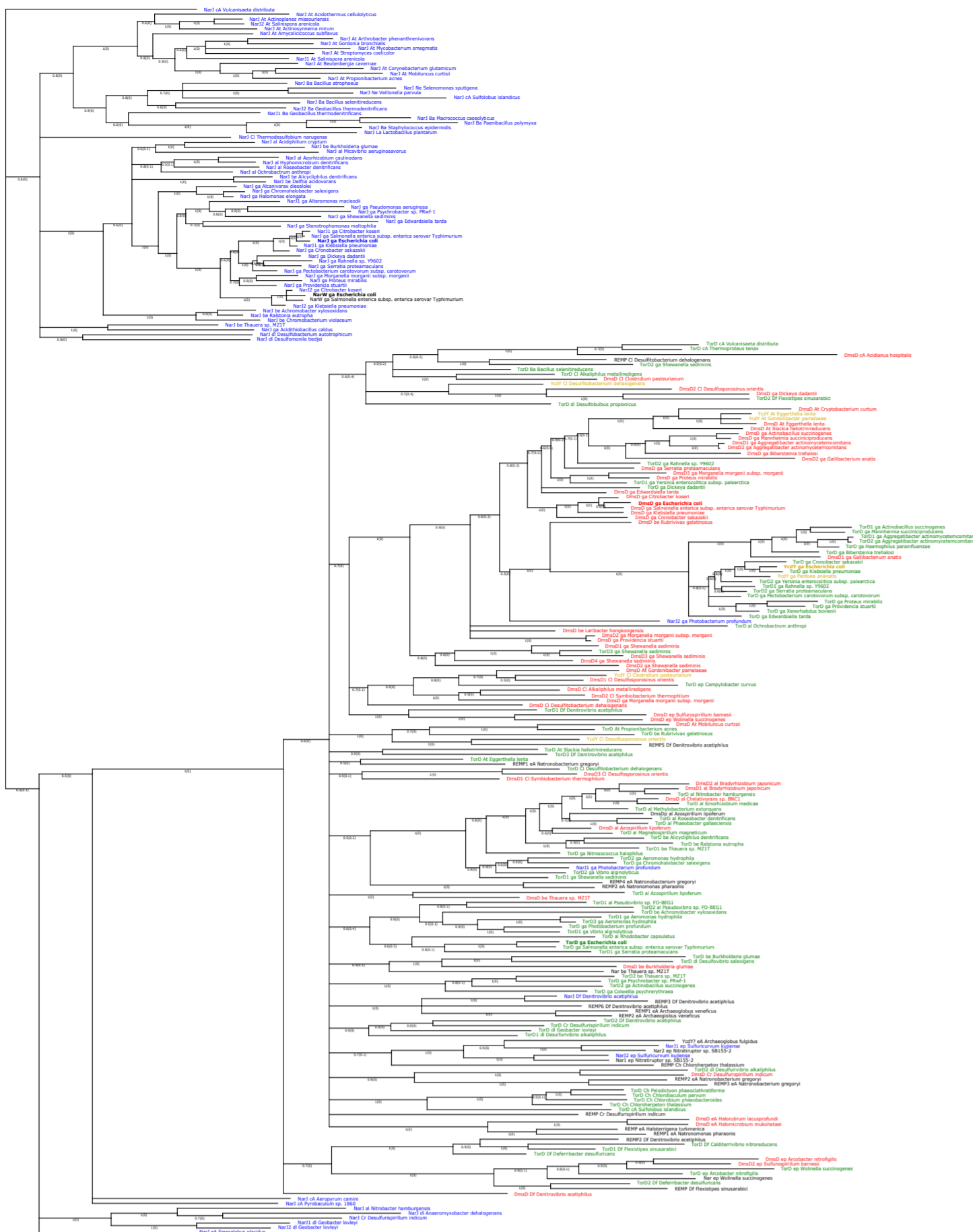

**NarJ-Bayes**

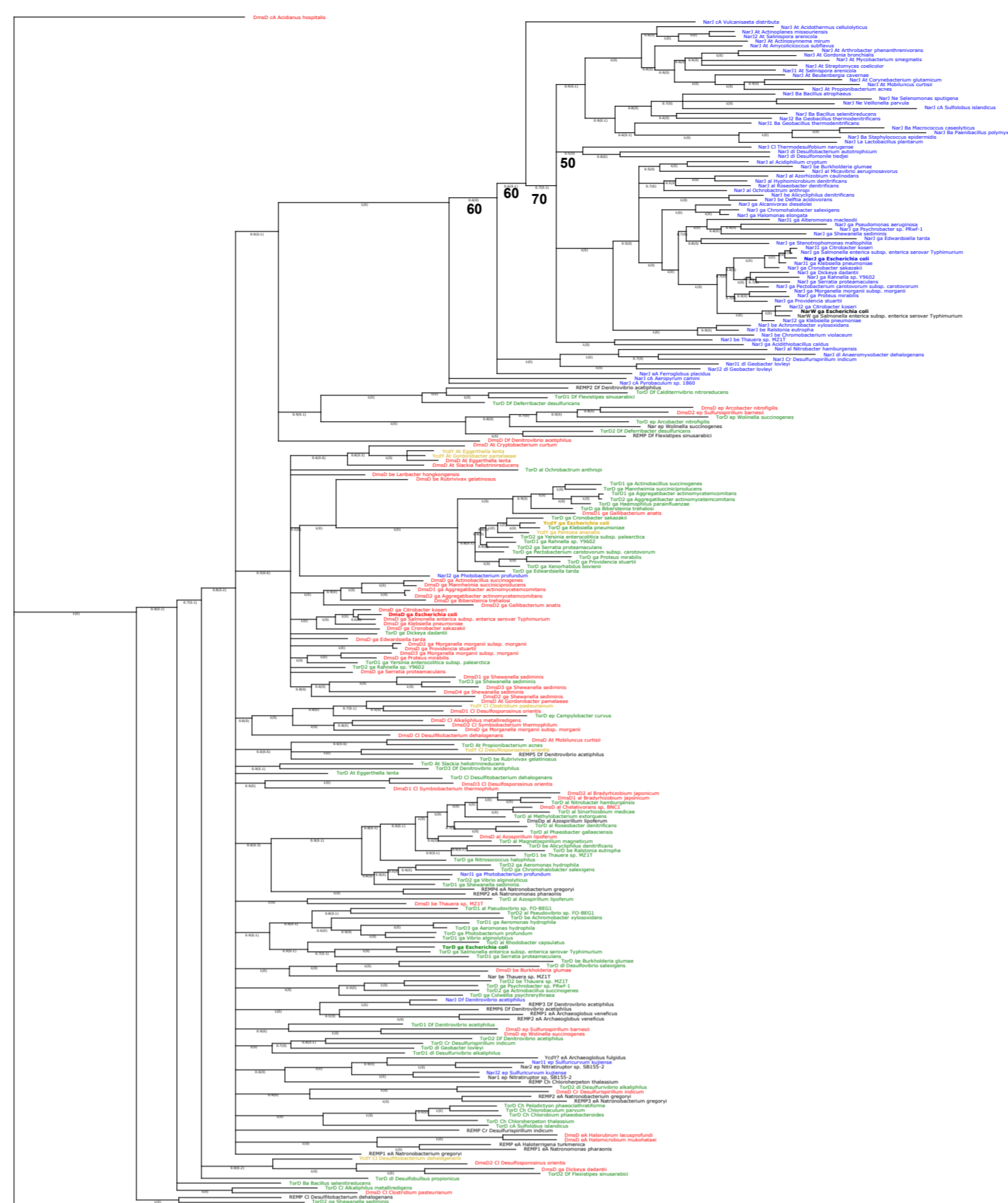

**DmsD-Bayes**

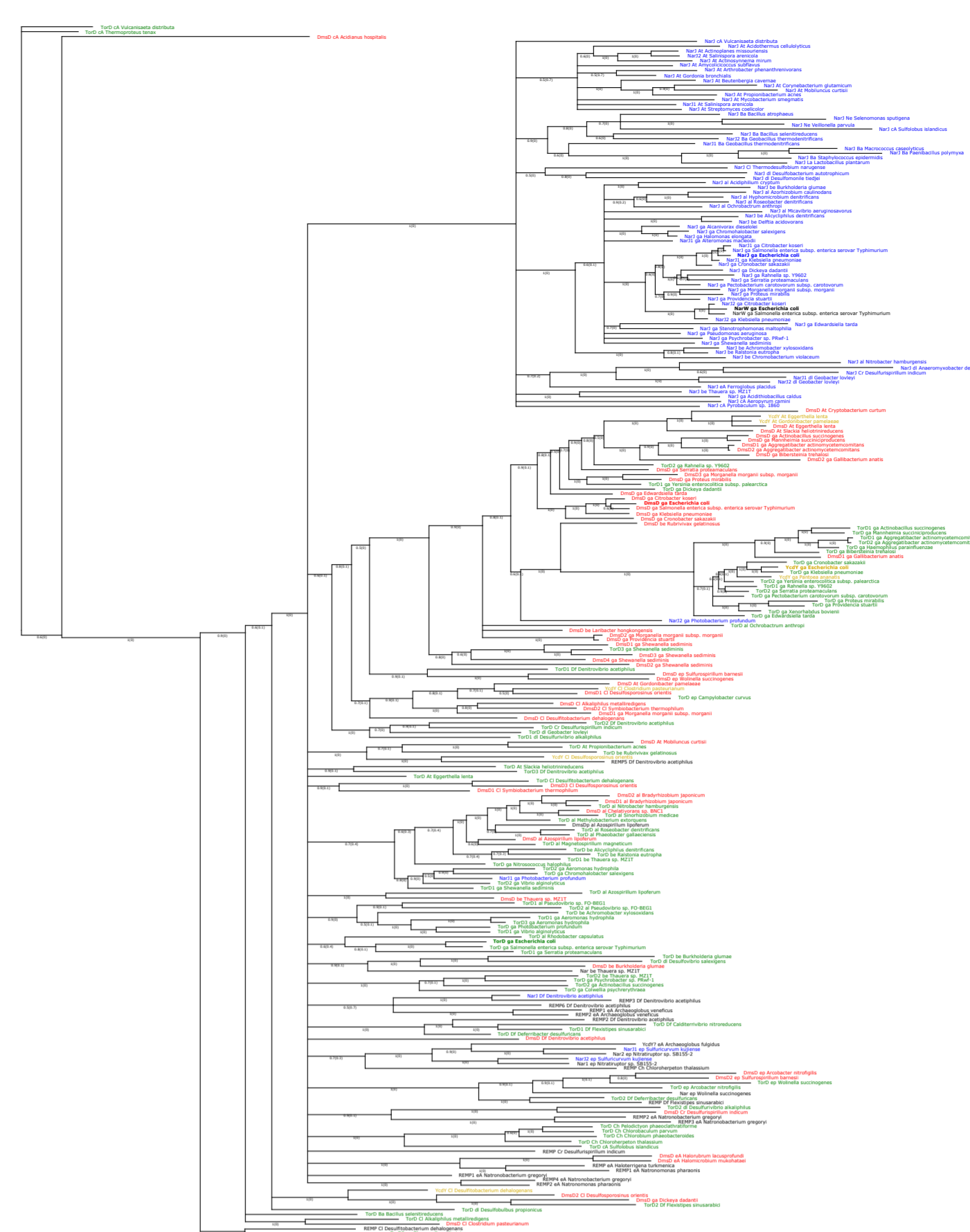

**TorD-Bayes**

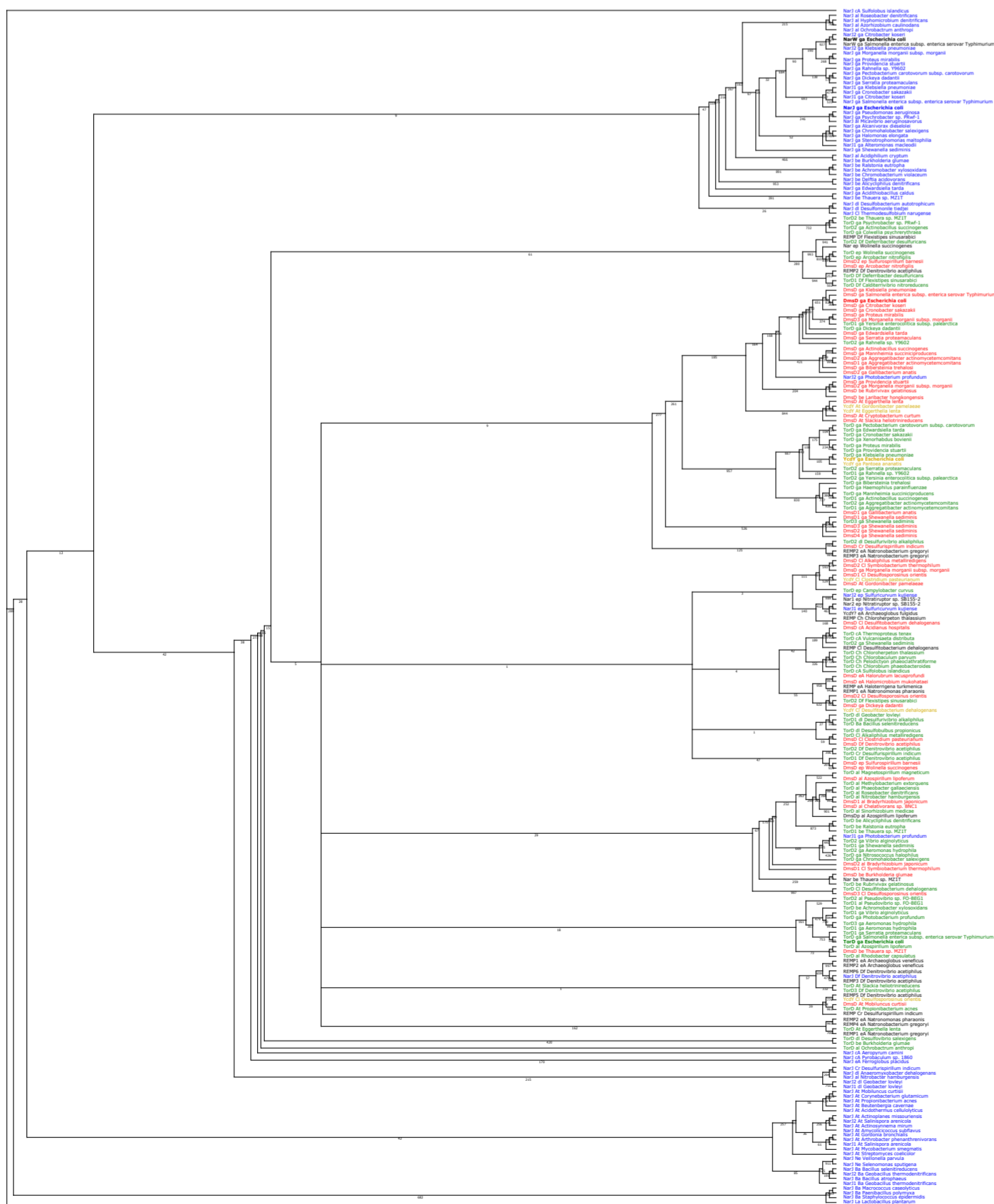

**NarJ-NJ**

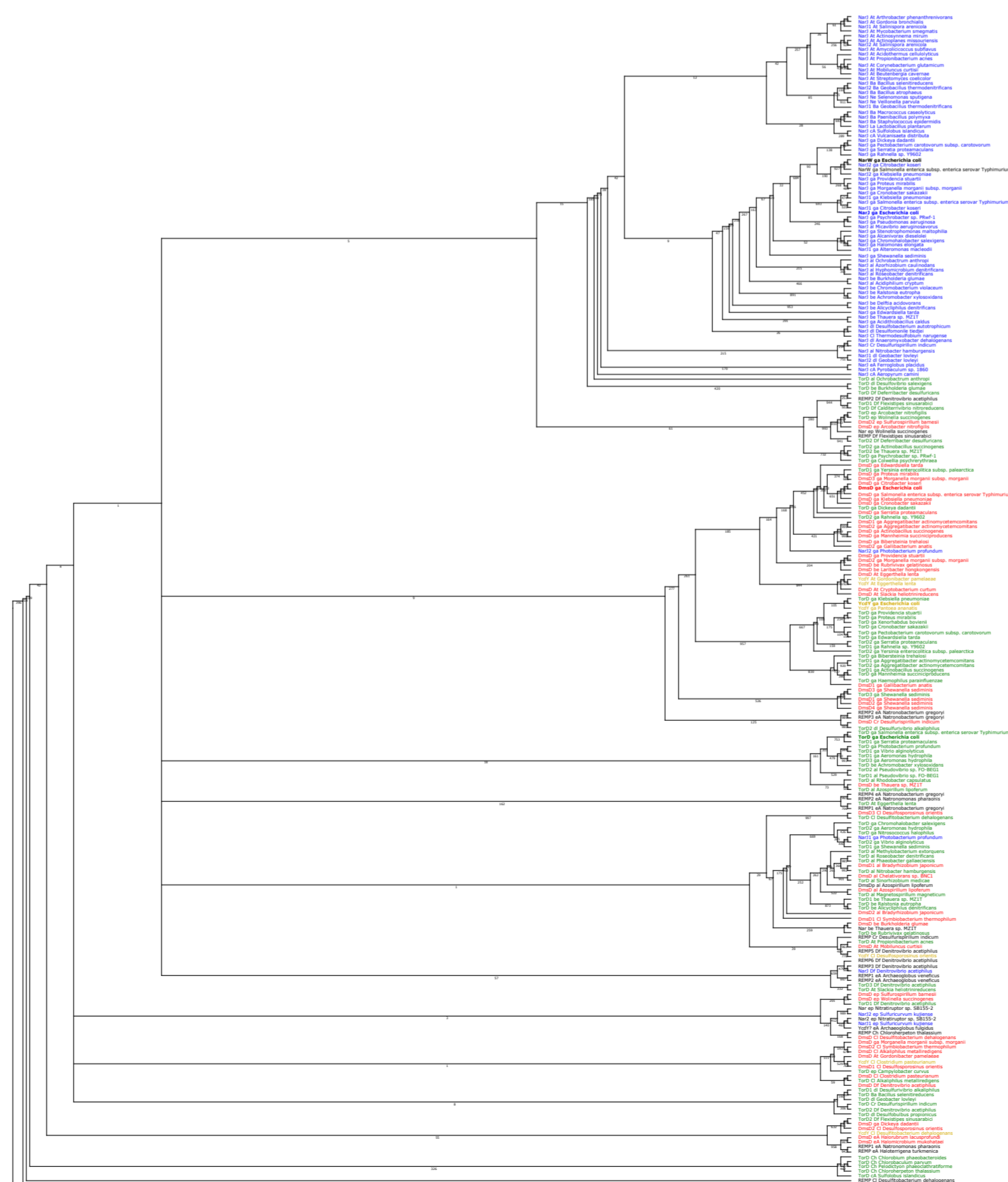

**DmsD-NJ**

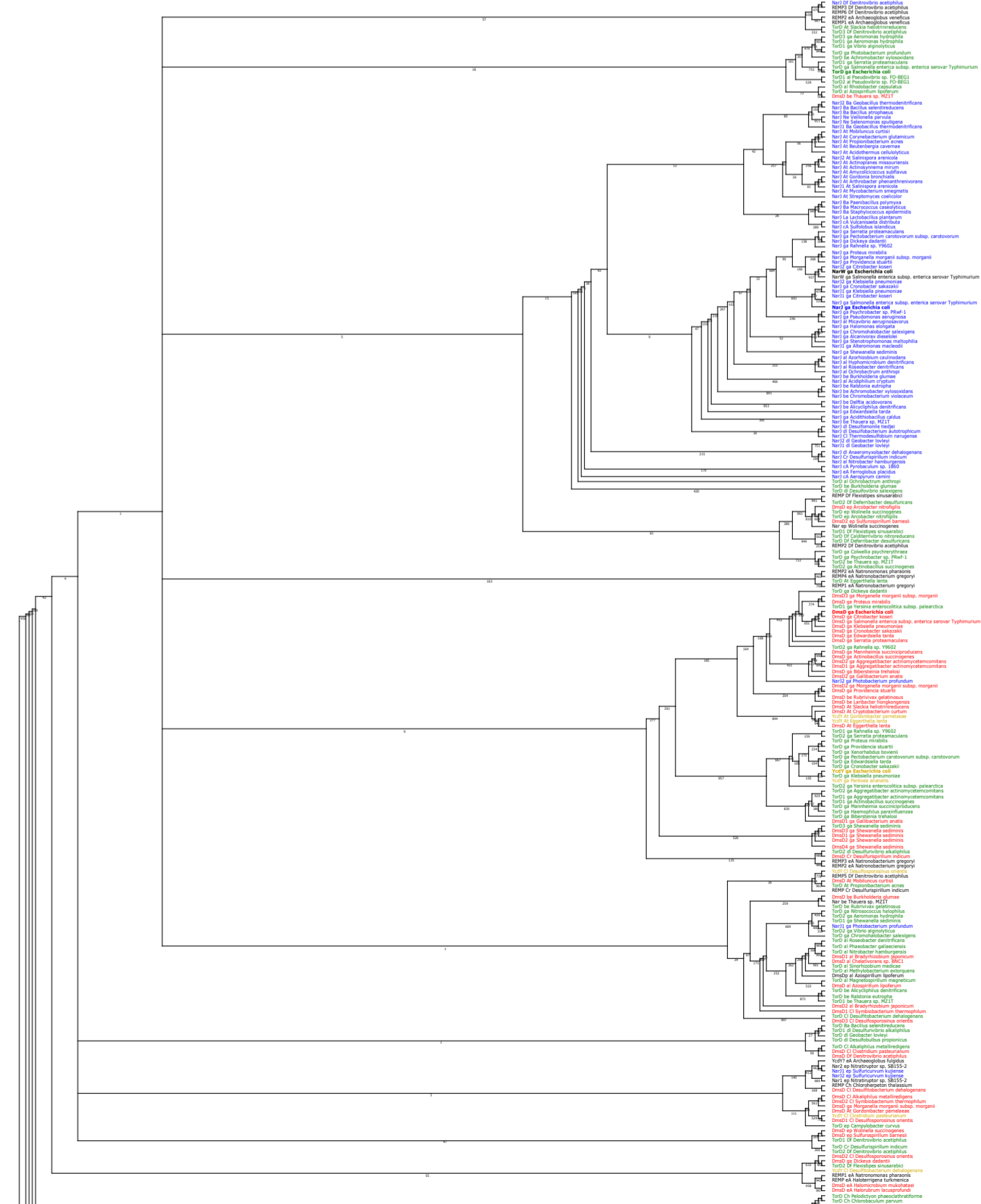

**TorD-NJ**

Supplement: Additional file 3: Figure S3. — Rooted BI and NJ phylograms of NarJ subfamily sequences generated using Crenarchaeal NarJ, DmsD or TorD sequences as outgroups. A total of 6 rooted phylograms of 324 protein sequences from 130 different Prokaryotic species is shown after NJ and BI analyses using Vulcanisaeta distributa NarJ, Thermoproteus tenax TorD or Vulcanisaeta distributa DmsD sequences as outgroups. For BI phylograms, 10 million generations were performed and posterior probability values (0–1) at each node of the dendrogram are shown to provide branch confidence. Rooted NJ phlyograms of 324 protein sequences were determined from a consensus of 1000 bootstrapped replicate trees and confidence values (as a total of 1000) are indicated beside each node in the dendrogram. In all panels the genus and species and NarJ subfamily annotation are listed on the right branches and coloured according to NarJ subfamily annotation (NarJ blue; DmsD red; TorD green; YcdY yellow). [file 12862_2015_412_MOESM3_ESM.pdf]

Figure S4

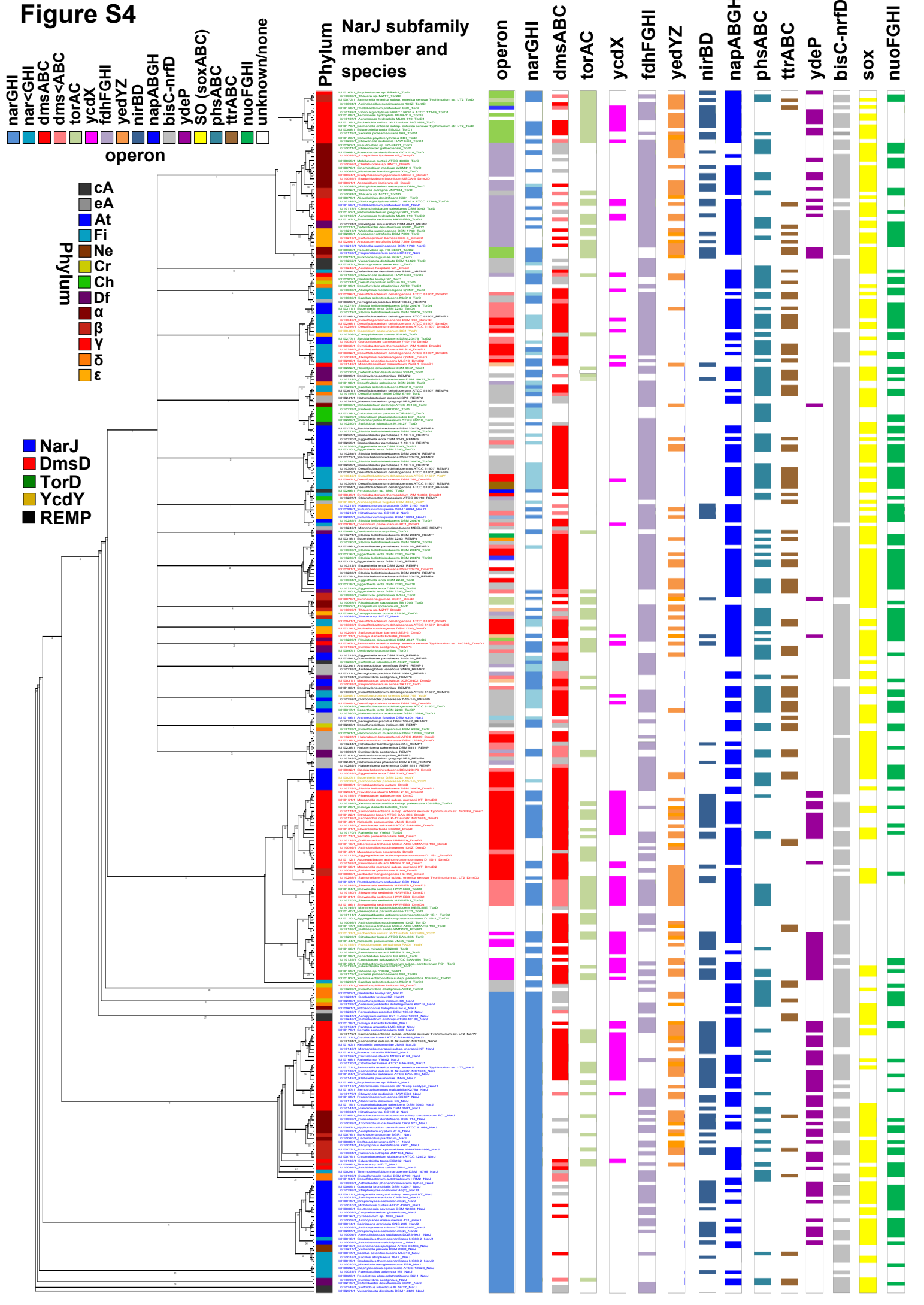

Supplement: Additional file 4: Figure S4. — The rooted NJ phylogram of 324 NarJ subfamily proteins and heatmap of their respective CISM operon associations. The Vulcanisaeta distributa NarJ rooted NJ phlyogram of 324 protein sequences was determined from a consensus of 1000 bootstrapped replicates and confidence values (as a total of 1000) are indicated beside each node in the dendrogram. Coloured heatmaps on the right-hand side of REMP Genus species labels indicate the operon association of the subfamily member, the presence of one or more CISM operons (narGHI, dmsABC, torAC and ycdX), and other CISMs indentified within each species genome (refer to legend in panel and CISM operon definitions provided in Additional file 6: Figure S2). [file 12862_2015_412_MOESM4_ESM.pdf]

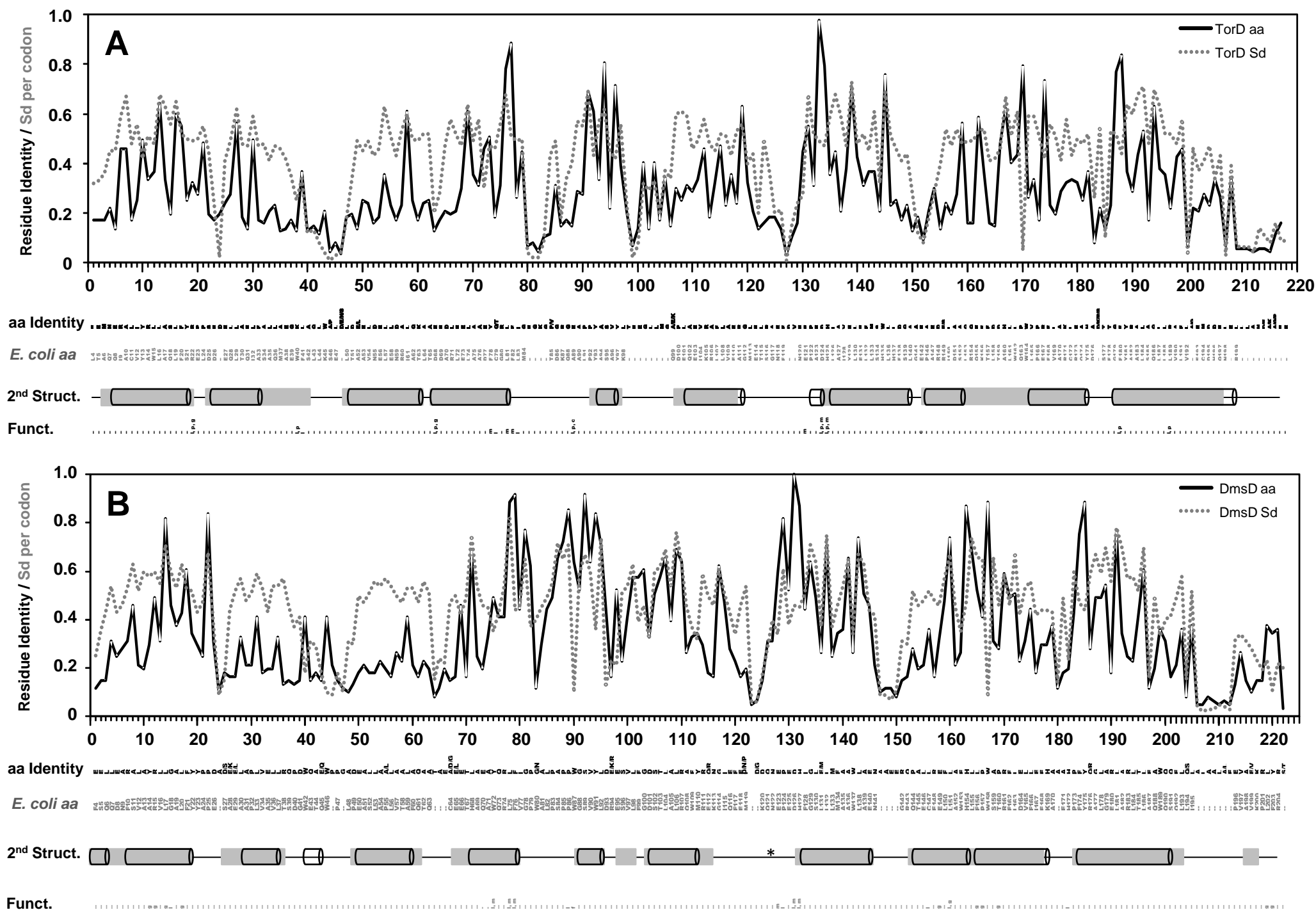

Figure S5. A-B

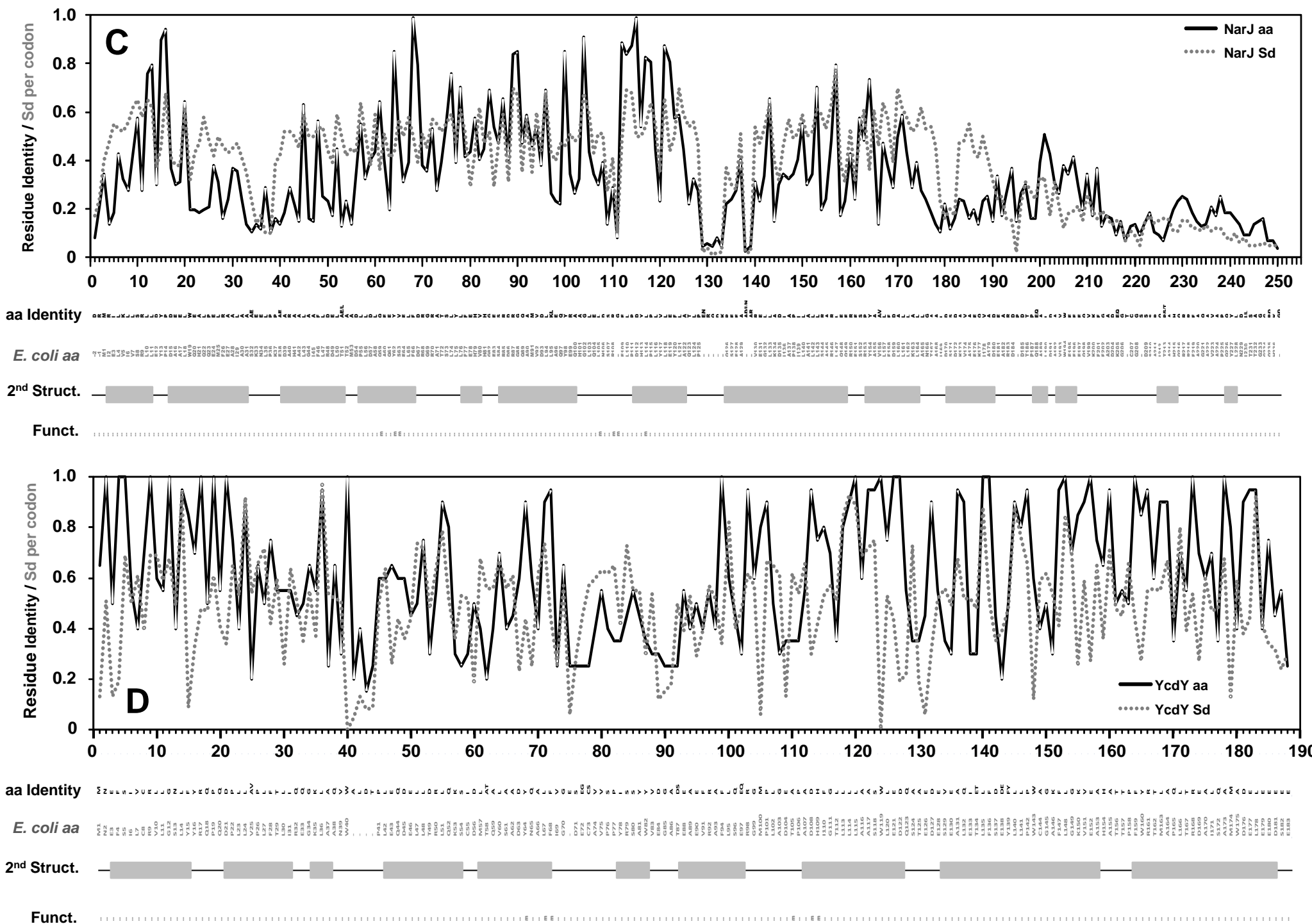

Figure S5. C-D

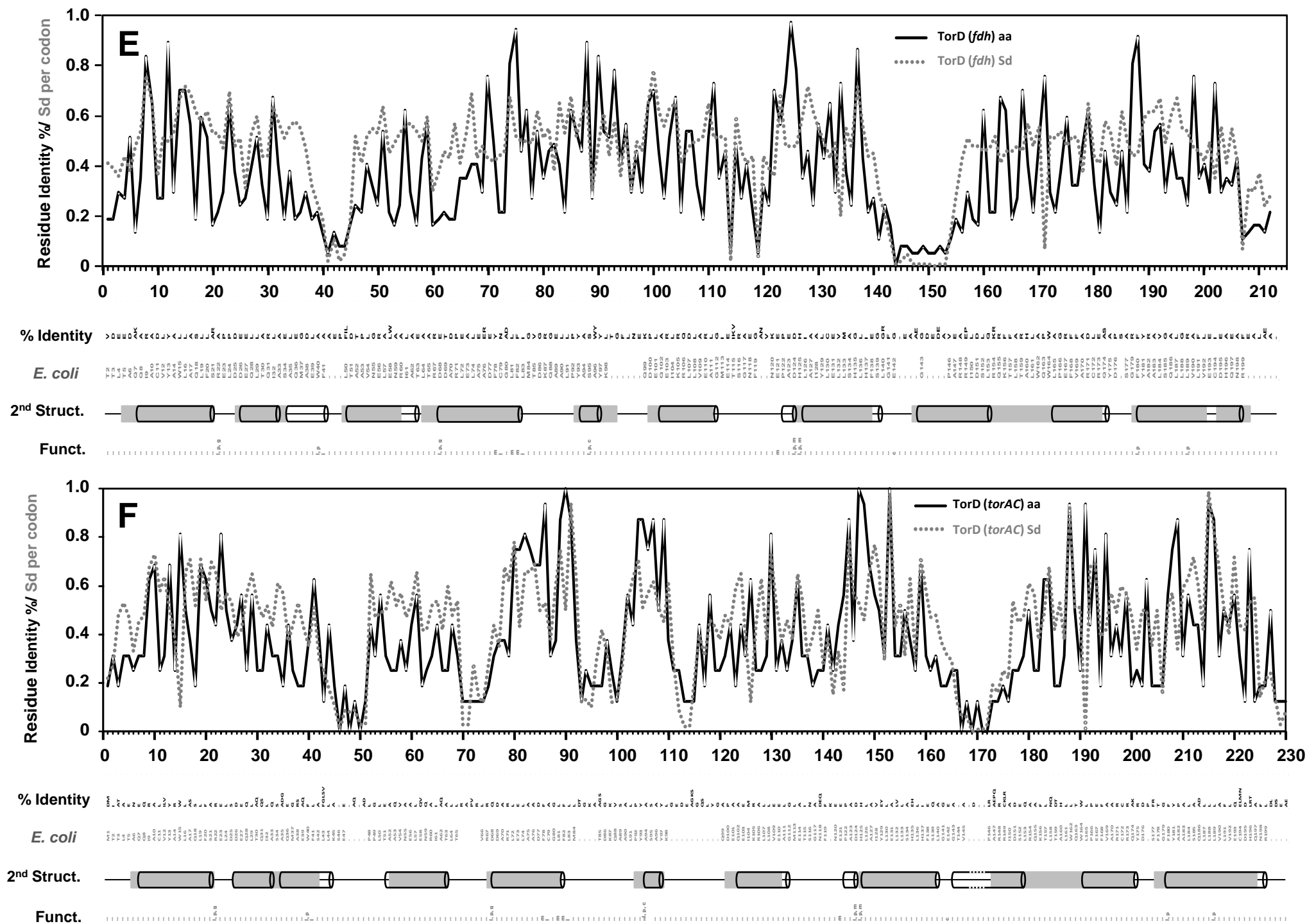

Figure S5. E-F

Supplement: Additional file 5: Figure S5. — A summary of NarJ subfamily amino acid percentage identities and Sd codon values determined from MSA and syn-scanning analysis. TorD residue % identity and Sd codon values for sequences that were genetically located within fdh operons (E) or torAC operons (F) are shown. Both charts show the residue identity (as a decimal value 0-1; black lines) and synonymous nucleotide rate (Sd) per codon (grey lines) that correspond to each numbered amino acid/ codon position (x-axis) in the overall NarJ subfamily alignment (in black font) or to the specified E. coli subfamily member (in grey font). Predicted secondary structures (α-helices; grey rectangles, and turns/loops; lines) are shown as a cartoon line diagram and α-helical secondary structure shown as cylinders correspond to the E. coli DmsD (panel B; 3EFP, 3CW0, 3U41 [50, 57, 60]) or S. massila TorD (panel A; 1N1C [61]) crystal structures below both residues alignments. Residues known to have experimental and/or predicted involvement in TAT leader/signal sequence binding (l), leader proofreading (p), co-factor insertion (c), GTP binding (g), folding stability (f), dimerization (d) and REMP motif (m) are provided below structural information. [file 12862_2015_412_MOESM5_ESM.pdf]

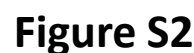

### Figure S2

Supplement: Additional file 6: Figure S2. — A summary heatmap of NarJ subfamily operons and their respective CISM complex genes detected in all 130 Prokaryotic species genomes. Genus and species are listed on the y-axis and grouped alphabetically by phylum (according to the color bar on the left hand y-axis). Each column indicates the presence (coloured) and absence (white/uncoloured) of one or more of the genes listed on the upper x-axis of the dendrogram. The top x-axis indicates the subfamily member-CISM operon associations (left-hand columns) and other CISM genes/ operons identified from genome searches of each species. NarJ subfamily members termed “other” refer to sequences annotated as ‘nitrate reductase delta’ and/or ‘REMP’. Definitions for abbreviated CISM operons are as follows: bisC-nrfD; biotin sulfoxide reductase (-hybA) -polysulfide reductase; fdhGHI; formate dehydrogenase, napABCHG; periplasmic nitrate reductase, nirBD; cytosolic nitrate reductase, nuoGHI; NADH:ubiquinone oxidoreductase I, phsABC; polysulfide reductase; sox; sulfoxide reductase (includes methionine sulfoxide reductases and sulfite oxidases), ttrABC; tetrathionate reductase, ydeP; periplasmic acid resistance protein/ formate dehydrogenase homologue, yedZY; putative MobisPD oxidoreductase. [file 12862_2015_412_MOESM6_ESM.pdf]
